# Intron turnover is essential to the development and pathogenicity of the plant pathogenic fungus *Fusarium graminearum*

**DOI:** 10.1038/s42003-022-04111-3

**Published:** 2022-10-26

**Authors:** Yejin Choi, Hyun-Hee Lee, Jiyeun Park, Sieun Kim, Soyoung Choi, Heeji Moon, Jiyoung Shin, Jung-Eun Kim, Gyung Ja Choi, Young-Su Seo, Hokyoung Son

**Affiliations:** 1 Department of Agricultural Biotechnology, Seoul National University, Seoul, 08826 Republic of Korea; 2 Department of Integrated Biological Science, Pusan National University, Busan, 46247 Republic of Korea; 3 Research Institute of Agriculture and Life Sciences, Seoul National University, Seoul, 08826 Republic of Korea; 4 Therapeutic & Biotechnology Division, Center for Eco-friendly New Materials, Korea Research Institute of Chemical Technology, Daejeon, 34114 Republic of Korea

**Keywords:** Fungal pathogenesis, Fungal genetics, Pathogens

## Abstract

Intron lariats excised during the splicing process are rapidly degraded by RNA lariat debranching enzyme (Dbr1) and several exonucleases. Rapid turnover of lariat RNA is essential to cellular RNA homeostasis. However, the functions of Dbr1 have not been investigated in filamentous fungi. Here, we characterized the molecular functions of Dbr1 in *Fusarium graminearum*, a major fungal plant pathogen. Deletion of *FgDBR1* resulted in pleiotropic defects in hyphal growth, conidiation, sexual reproduction, and virulence. Through transcriptome analysis, we revealed that the deletion mutant exhibited global accumulation of intron lariats and upregulation of ribosome-related genes. Excessive accumulation of lariat RNA led to reduced overall protein synthesis, causing various phenotypic defects in the absence of *FgDBR1*. The results of this study demonstrate that a compromised intron turnover process affects development and pathogenesis in this fungus and that Dbr1 function is critical to plant pathogenic fungi.

## Abstract (web summary)

RNA lariat debranching enzyme Dbr1 is required for intron turnover in the fungal plant pathogen <i>Fusarium graminearum <i > , and accumulation of lariat RNA affects its development and pathogenesis.

## Introduction

In eukaryotic cells, introns in precursor mRNA (pre-mRNA) are excised during the splicing process to form mature mRNA. RNA splicing is a two-step reaction catalyzed by the spliceosome, a large RNA-protein complex^1,2^. In the first step, an adenosine residue at the branch site attacks the 5′ splice site. This reaction results in the production of a cleaved 5′ exon and an intron-3′ exon intermediate containing an unusual 2′-5′ phosphodiester bond. In the second reaction, the 3′ hydroxyl group of the 5′ exon attacks the 3′ splice site, resulting in the release of the intron lariat and ligation of two exons, thereby forming mature mRNA^3^. Then, the excised intron lariat is rapidly degraded in the intron turnover pathway. RNA lariat debranching enzyme Dbr1, which cleaves the 2′-5′ phosphodiester bond, linearizes the lariat intron into a linear molecule that is further degraded or processed by exoribonucleases^4^. This rapid turnover of introns is important for normal cellular functions, including maintenance of RNA homeostasis, in eukaryotes^5^.

The gene encoding Dbr1 was originally identified and cloned from the budding yeast *Saccharomyces cerevisiae* in a study to identify the host cellular factors involved in the transposition of the Ty1 retroelement^4^. The *S. cerevisiae dbr1* null mutant showed reduced Ty1 transposition frequency and increased accumulation of intron lariats, indicating that lariat debranching is a rate-limiting step in the intron degradation pathway. Although deletion of *DBR1* had little effect on the growth of *S. cerevisiae*, a *dbr1* null mutant of the fission yeast *Schizosaccharomyces pombe* exhibited not only high-level accumulation of intron lariats but also severe growth defects^6^. In addition, the human pathogen *Cryptococcus neoformans* showed reduced virulence when *DBR1* was deleted^7^. In *Arabidopsis* and humans, *dbr1*-deletion mutants showed embryonic lethality^8,9^ and their orthologues of *DBR1* complemented the mutant phenotypes of yeast *dbr1* null mutants^10^. Taken together, these findings demonstrate that the biochemical function of Dbr1 is well conserved evolutionarily, but rapid intron turnover appears to be more important in higher eukaryotes that contain numerous introns.

The ascomycete fungus *Fusarium graminearum* is an important plant pathogen that is the major causal agent of Fusarium head blight (FHB) in various cereal crops worldwide^11^. Epidemics of FHB cause severe yield losses and contamination of grains with mycotoxins such as trichothecenes and zearalenone, which pose risks to humans and animals^12^. Although several biological and chemical methods have been proposed to control FHB, they have not effectively prevented disease incidence. Therefore, a comprehensive understanding of the cellular and molecular bases of fungal development and pathogenesis is required for the establishment of efficient and sustainable strategies to manage FHB.

Although *DBR1* orthologues are well conserved in eukaryotes, their pathobiological role in filamentous pathogenic fungi remains unclear. Thus, the objectives of this study were to characterize the molecular function of *DBR1* and investigate the importance of the intron turnover process in *F. graminearum* through a combination of genetic and transcriptomic approaches. Our data demonstrated that the biochemical function of FgDbr1 was conserved and required for vegetative growth, asexual and sexual development, and virulence in this fungus. Using RNA-sequencing (RNA-seq) analysis, we revealed that phenotypic defects of the deletion mutant were driven by genome-wide accumulation of intron lariats and a consequent decrease in translational efficiency. To our knowledge, this study is the first to characterize a lariat debranching enzyme in filamentous fungi and provides strong evidence that the intron turnover process is important for development and pathogenesis in *F. graminearum*.

## Results

### Identification of the RNA lariat debranching enzyme FgDbr1 in *F. graminearum*

**Fig. 1:**
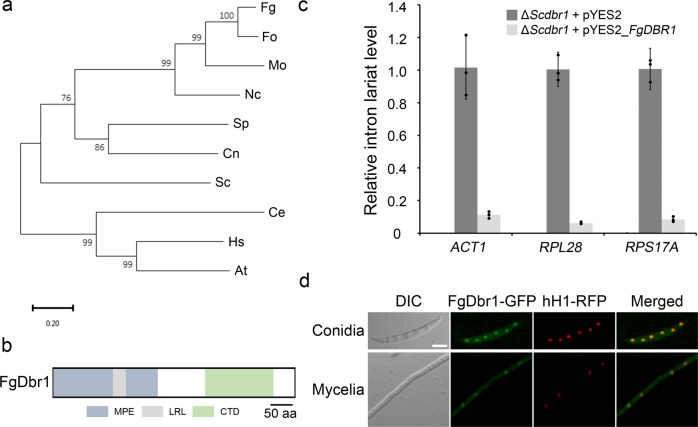
Characterization of the intron lariat debranching enzyme FgDbr1. **a** Phylogenetic tree of Dbr1 homologues. Amino acid sequences were aligned using the ClustalW, and MEGA X software was used to perform phylogenetic analysis using the neighbor-joining method with 1000 bootstrap replicates. Fg *Fusarium graminearum*, Fo *Fusarium oxysporum*, Mo *Magnaporthe oryzae*, Nc *Neurospora crassa*, Sp *Schizosaccharomyces pombe,* Cn *Cryptococcus neoformans,* Sc *Saccharomyces cerevisiae,* Ce *Caenorhabditis elegans,* Hs *Homo sapiens*, At *Arabidopsis thaliana*. **b** Domain architecture of FgDbr1. MPE domain including LRL motif is conserved in FgDbr1. MPE methallophosphoesterase domain, LRL lariat recognition loop, CTD C-terminal domain. **c** Heterologous expression of FgDBR1 in yeast. Relative levels of intron lariat were quantified by quantitative real-time PCR (qRT-PCR) in the mutant containing empty vector pYES2 or pYES2-FgDBR1. Relative level of intron lariat in the wild-type BY4741 containing empty vector pYES2 was not detectable. Error bars indicated standard deviations of three biological replicates. ACT1, RPL28 and RPS17A represent introns of ACT1, RPL28 and RPS17A genes in *S*. *cerevisiae*, respectively. **d** Subcellular localization of FgDbr1. FgDbr1 was fused with green fluorescent protein (GFP) and histone H1 with red fluorescent protein (RFP). The yellow color in the merged images indicates co-localization. Scale bar = 10 μm.

A BLASTp search was performed using *S. cerevisiae* Dbr1 as a query against the *F. graminearum* genome database. We identified a Dbr1 homologue in *F. graminearum* at the FGRAMPH1_01G00807 locus that encoded 544 amino acids and was designated as *FgDBR1*. FgDbr1 shares 35% and 40% overall amino acid sequence identity with homologues in *S. cerevisiae* and *S. pombe*, respectively. Phylogenetic analysis indicated that Dbr1 is highly conserved in eukaryotic organisms and that FgDbr1 is closely related to the Dbr1 proteins of filamentous fungi (Fig. 1a). Furthermore, domain analysis and sequence alignment showed that FgDbr1 contains a highly conserved N-terminal domain belonging to the metallophosphoesterase (MPE) superfamily and a C-terminal domain lacking detectable sequence or structural homology to any other class of enzymes (Fig. 1b). Additionally, the protein contains a conserved amino acid motif (GNHE), which is essential to its debranching activity, and a lariat recognition loop (LRL), which is a unique feature of Dbr1 not found in other MPEs (Supplementary Fig. 1a)^13^.

To validate the intron lariat debranching activity of FgDbr1, we introduced full-length *FgDBR1* cDNA into a *DBR1* deletion mutant of *S. cerevisiae* BY4741 (Δ*Scdbr1*) using a yeast expression vector (pYES2). Because the Δ*Scdbr1* mutant showed increased accumulation of intron lariats, we analyzed intron lariat RNA levels through quantitative real-time PCR (qRT-PCR) in each yeast strain. *FgDBR1* complemented the defective intron lariat accumulation phenotype of the yeast deletion mutant, supporting the intron lariat debranching activity of FgDbr1 (Fig. 1c).

### Subcellular localization of FgDbr1

Because pre-mRNA splicing occurs in the nucleus releasing intron lariats as byproducts, we generated the FgDBR1c-r strain (Δ*Fgdbr1::FgDBR1-GFP-HYG hH1::hH1-RFP-GEN*) by an outcross between the mat1r^14^ and FgDBR1c strains to determine the nuclear localization of FgDbr1. Although FgDbr1-GFP was observed in both the nuclei and cytoplasm of the FgDBR1c-r strain, it was mainly localized in the nuclei (Fig. 1d). This result is consistent with the previous finding that Dbr1 is a nucleocytoplasmic shuttling protein in humans and *Arabidopsis*^15,16^.

### *FgDBR1* is important for vegetative growth and conidiation

**Fig. 2:**
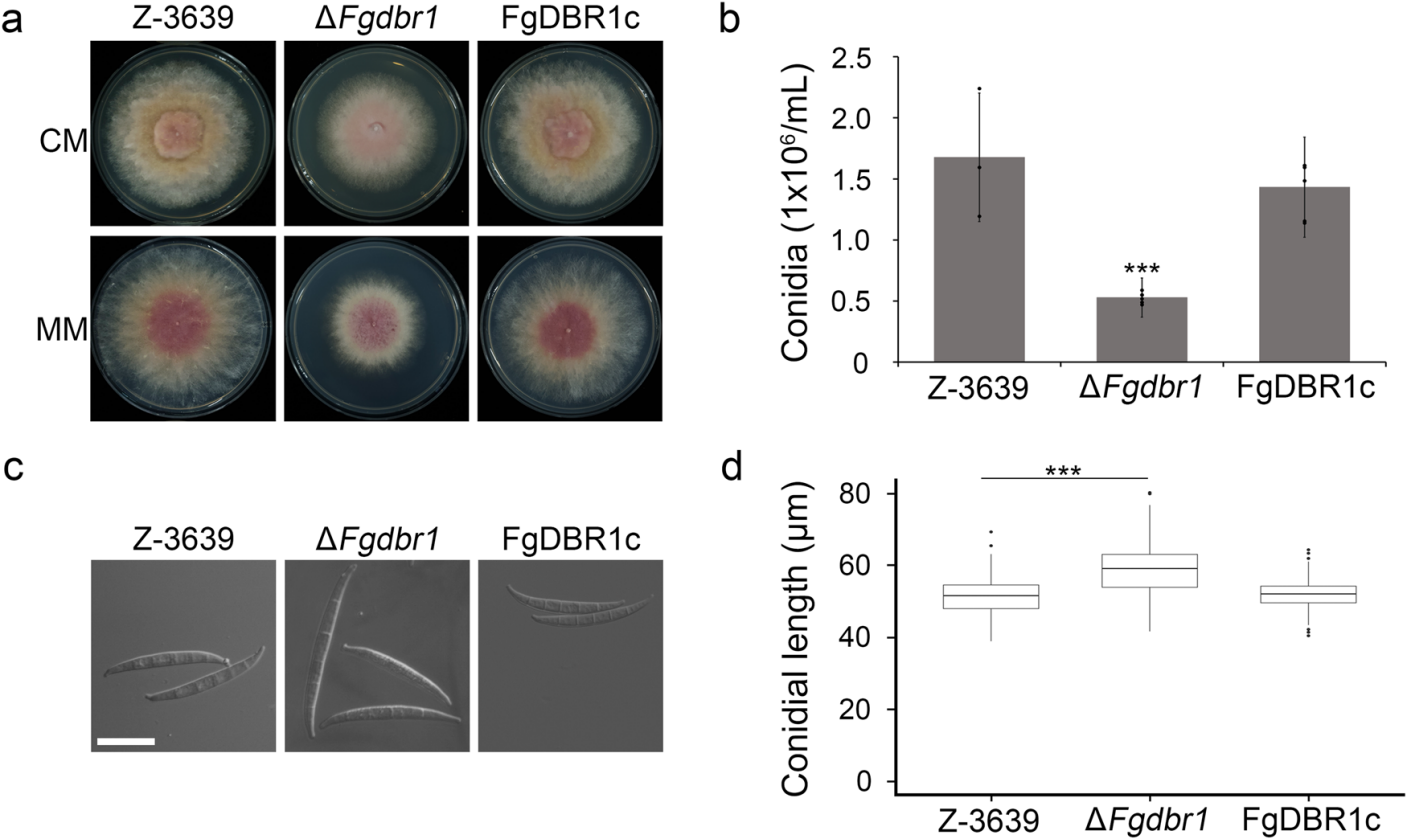
Vegetative growth and conidiation of *F. graminearum* strains. **a** Mycelial growth of *F. graminearum* strains on complete medium (CM) and minimal medium (MM). Pictures were taken 5 days after inoculation. **b** Conidial production. The number of conidia generated by each strain was counted after 5 days of incubation in carboxymethyl cellulose (CMC) medium. Error bars represent standard deviations of biological replicates (*n* = 3 for wild type, *n* = 6 for Δ*Fgdbr1* and FgDBR1c). The triple asterisks indicate significant difference with *p* < 0.001. **c** Conidial morphology. Conidia were induced on yeast malt agar (YMA) for 48 h and subsequently observed by differential interference contrast (DIC) microscopy. Scale bar = 25 μm. **d** Boxplot showing conidial length of each strain. Lower and upper whiskers represent 1.5 times of the lower and upper interquartile ranges (IQR), respectively, and median values were indicated by solid black lines. The length of 300 conidia induced on YMA were measured for each strain. ***p < 0.001.

Although Dbr1 is a highly conserved protein in eukaryotes, the phenotypes caused by deletion of the Dbr1-encoding gene vary among species. To investigate the physiological functions of FgDbr1 in *F. graminearum*, we generated deletion mutants by replacing *FgDBR1* with a geneticin resistance gene cassette (*GEN*) in the wild-type strain. Southern blot hybridization was used to confirm the deletion of *FgDBR1* (Supplementary Fig. 2). Furthermore, the *FgDBR1* open reading frame (ORF) was fused to green fluorescent protein (*GFP*) under its native promoter and introduced into the Δ*Fgdbr1* strain to produce a complementation strain (FgDBR1c). On complete medium (CM) and minimal medium (MM), we compared vegetative growth among the wild type, Δ*Fgdbr1*, and the complemented strains. The Δ*Fgdbr1* mutants showed slightly reduced growth rates compared to the wild-type and complemented strains (Fig. 2a and Table 1), whereas Δ*Scdbr1* showed no growth difference from the wild-type strain (Supplementary Fig. 1b).

**Table 1:** Phenotypic characterization of the Δ*Fgdbr1* mutant.

Strain	Colony diameter (cm)^a^		Conidiation (×10^6^/mL)^b^	Conidia (µm)^c^			Virulence (disease index)^d^
	CM	MM		Length	Width	Septa	
Z-3639	7.96 ± 0.09	8.36 ± 0.05	1.68 ± 0.53	51.1 ± 5.6	5.0 ± 2.3	4.6 ± 0.6	4.4 ± 1.9
Δ*Fgdbr1*	6.89 ± 0.08***	6.10 ± 0.12***	0.53 ± 0.16***	58.8 ± 7.7***	4.5 ± 0.5***	4.3 ± 0.9***	1.3 ± 0.4***
FgDBR1c	7.99 ± 0.11	8.22 ± 0.08	1.43 ± 0.41	52.0 ± 3.8	4.7 ± 2.7	4.7 ± 0.5*	4.8 ± 1.8

^a^Colony diameter was measured after 5 days of incubation.

^b^Conidiation was measured by counting the number of conidia produced after 5 days of incubation in CMC medium.

^c^Macroconidia were produced on YMA. In total, 300 macroconidia were examined.

^d^Disease index (number of diseased spikelets per wheat head) for each strain was measured at 21 days after inoculation.

Single asterisk represents a significant difference at *p* < 0.05 according to *t*-test.

Triple asterisks represent significant differences at *p* < 0.001 according to *t*-test.

To determine whether *FgDBR1* is required for asexual development, each strain was cultured on carboxymethylcellulose (CMC) medium. Conidial production was reduced dramatically in the Δ*Fgdbr1* mutants compared to the wild-type strain (Fig. 2b and Table 1). Moreover, deletion of *FgDBR1* resulted in the production of abnormally shaped conidia. Conidia of the Δ*Fgdbr1* mutants exhibited increased length and greater variability compared to those of the wild type (Fig. 2c, d, and Table 1). The phenotypic defects in Δ*Fgdbr1* mutants were restored in the complementation strain, FgDBR1c. These results indicate that *FgDBR1* is required for vegetative growth and asexual reproduction in *F. graminearum*.

### *FgDBR1* is essential for sexual reproduction and virulence

**Fig. 3:**
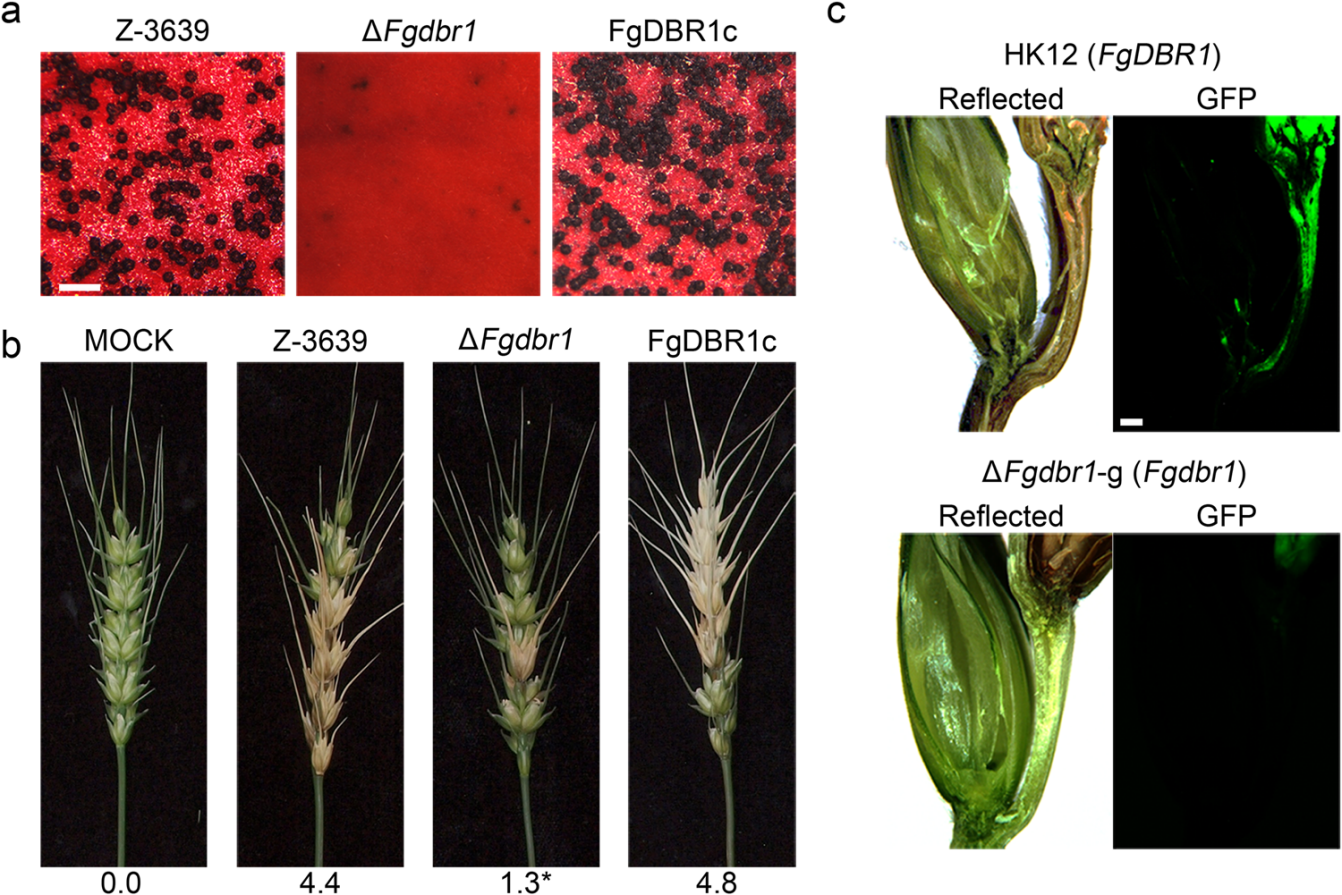
Sexual development and virulence of *F*. *graminearum* strains. **a** Sexual development on carrot agar medium. A 5-day-old culture in carrot agar was mock fertilized to induce sexual reproduction. Pictures were taken 7 days after sexual induction. Scale bar = 500 μm. **b** Virulence on wheat heads. The center spikelet of each wheat head was injected with 10 μl of conidial suspension. The pictures were taken 21 days after inoculation. The diseased index (diseased spikelets per wheat head) is denoted below the picture and the asterisks indicate significant difference with *p* < 0.05. Mock, mock inoculation was performed with 0.01% Tween 20. *n* = 15 and *n* = 10 independently inoculated spikelets for wild type and mutant strains, respectively. **c** Micrographs of manually generated sections after infection of wheat. Wheat spikelets were inoculated with conidial suspensions of cytosolic GFP-expressing strains (HK12 and Δ*Fgdbr1*-g) Infected wheat heads were longitudinally dissected at 6 days after inoculation and observed by fluorescence microscopy. GFP fluorescence signal indicates spreading of hyphae from the inoculation points. Arrowheads represent the inoculated spikelets. Reflected, bright field image. Scale bar = 500 μm.

Because ascospores serve as primary inocula in *F. graminearum* infection, we observed sexual reproduction. Seven days after sexual induction, Δ*Fgdbr1* mutants produced few immature perithecia, while the wild-type and complemented strains produced mature perithecia and asci containing eight ascospores (Fig. 3a).

The virulence of each strain was evaluated through point inoculation on flowering wheat heads. The wild-type and complemented strains caused typical head blight symptoms by 21 days after inoculation. The deletion mutants were able to colonize the inoculated spikelet, but failed to spread to adjacent spikelets on the head (Fig. 3b). Additionally, we generated a Δ*Fgdbr1*-g strain (Δ*Fgdbr1::GEN GFP-HYG*) via introduction of the pIGPAPA vector^17^ into the Δ*Fgdbr1* mutant to visualize mycelial movement on wheat heads during infection. At 6 days after inoculation, hyphae of HK12 strains, which carry the wild-type allele of *FgDBR1* and constitutively express cytosolic GFP, had spread to adjacent spikelets from the inoculated spikelet through rachis nodes. Δ*Fgdbr1*-g strains colonized only the inoculation points and failed to spread to neighboring spikelets (Fig. 3c).

As Δ*Fgdbr1* mutants were not able to spread infection over the wheat head, we examined the production of trichothecenes, which are virulence factors of *F. graminearum*^18^. The deletion mutants produced slightly lower levels of deoxynivalenol and 15-acetyldeoxynivalenol than did the wild type (Supplementary Fig. 3a). However, the transcript levels of trichothecene biosynthetic genes (*TRI5* and *TRI6*) did not differ significantly between the Δ*Fgdbr1* and wild-type strains (Supplementary Fig. 3b). Taken together, these data demonstrate that *FgDBR1* is indispensable for sexual reproduction and virulence in *F. graminearum*.

### Sensitivity of Δ*Fgdbr1* mutants to various stress conditions

Previous study showed that deletion of *DBR1* affected sensitivity to various stress agents in the basidiomycete fungus *C. neoformans*^7^. Therefore, to determine whether *FgDBR1* is required for stress responses in *F. graminearum*, we examined the responses of the deletion mutants to various environmental stresses. The tested conditions included reactive oxygen species (ROS), osmotic (or ionic), cell-wall (or membrane), and acidic (pH **=** 4) and basic (pH **=** 11) stresses, and several fungicide treatments (Supplementary Figs. 4a and 5). Additionally, we investigated sensitivity to DNA-damaging agents (Supplementary Fig. 4b). The Δ*Fgdbr1* mutants exhibited no significant differences in sensitivity to osmotic stress (Supplementary Fig. 5b) and increased resistance to cell-wall stress and acidic and basic conditions compared to the wild-type strain (Supplementary Fig. 5d, e). Notably, under various conditions—including oxidative, membrane and DNA damage stresses, as well as all fungicide treatments except for tebuconazole—mycelial growth was inhibited more severely in the deletion mutants than in the wild type (Supplementary Fig. 5b–d, f). These results indicate that *FgDBR1* is involved in general stress responses, especially in responses to damage to cellular macromolecules.

### Genome-wide accumulation of intron lariat RNA in the absence of *FgDBR1*

**Fig. 4:**
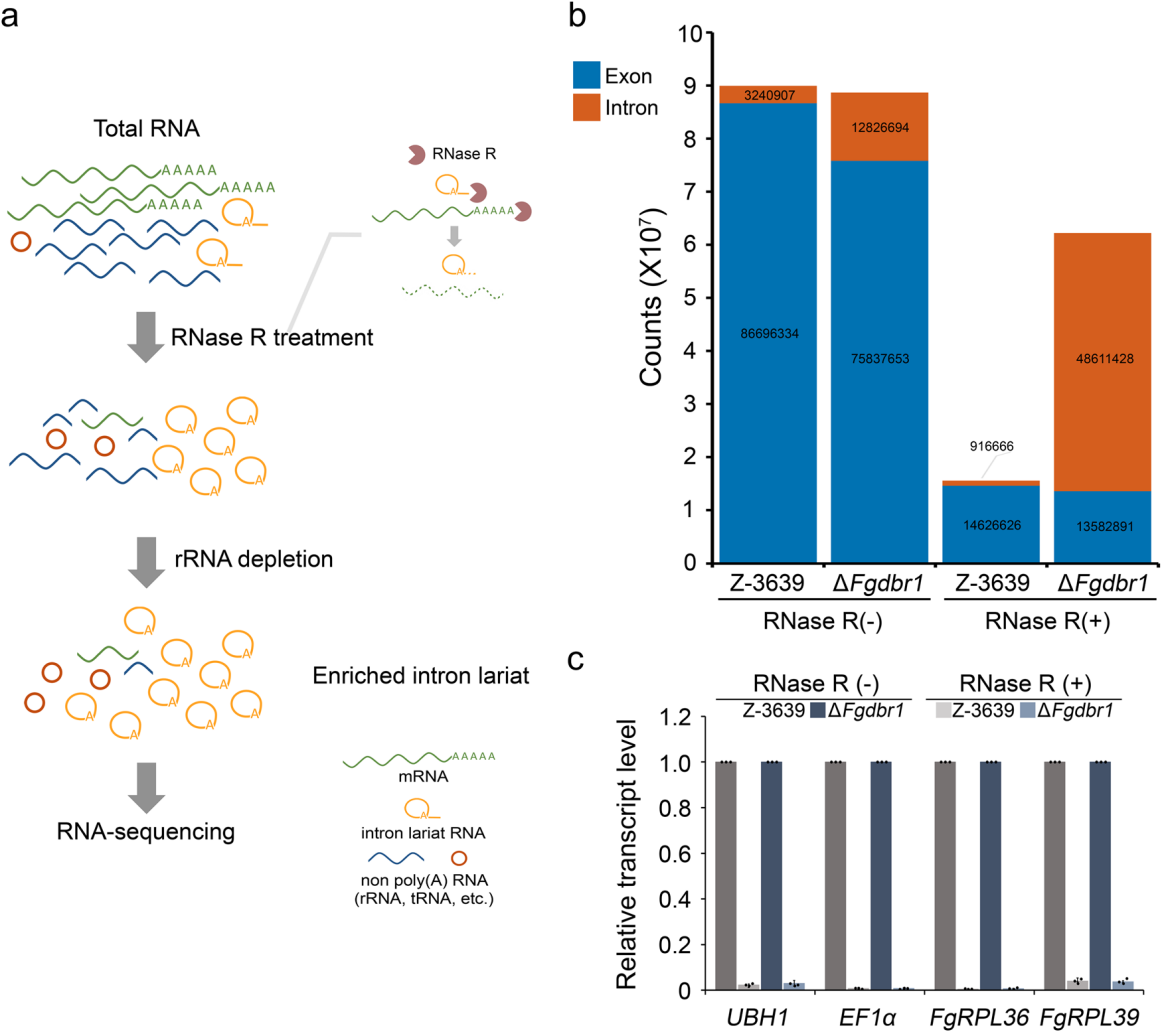
Transcriptome analysis of RNase R- treated or untreated samples in *F*. *graminearum* strains. **a** Workflow of intron lariat enrichment for RNA sequencing (RNA-seq). Total RNA was extracted from each strain grown for 24 h in liquid CM and digested with RNase R for linear RNA degradation. The remaining RNAs were further subjected to rRNA depletion and subsequently used for RNA-seq library construction. **b** Distribution of mapped read counts originating from exonic and intronic regions. **c** Verification of the elimination of linear RNA by RNase R. Relative transcript levels of the wild-type and Δ*Fgdbr1* samples with or without RNase R treatment were determined by qRT-PCR analysis. Error bars represent standard deviations of three biological replicates.

Given that the Δ*Fgdbr1* mutants exhibited defective phenotypes in various developmental stages, we performed transcriptome profiling of the wild-type and Δ*Fgdbr1* strains using RNA-seq to investigate the relationship between the accumulation of lariat RNA and fungal development. To examine the extent of intron lariat accumulation in Δ*Fgdbr1* efficiently, we conducted lariat RNA enrichment from total RNA using ribonuclease R (RNase R), which can degrade highly structured RNA molecules, including ribosomal RNAs (rRNAs) and linear RNAs, while retaining the loop portion of lariat RNA^19^ (Fig. 4a).

The total read counts mapped to exonic regions were 14626626 (41%) and 13582891 (24%) in RNase R-treated samples, and 86696334 (90%) and 75837653 (82%) in RNase R-untreated samples of the wild type and Δ*Fgdbr1*, respectively (Fig. 4b). Although the read counts from exonic regions in the wild-type and Δ*Fgdbr1* strains were similar, the exonic reads of the RNase R-treated sample were significantly reduced compared to the untreated sample. In the Δ*Fgdbr1* mutant, the proportion of intronic reads was significantly elevated in the RNase R-treated samples compared to the untreated samples. Furthermore, the RNase R-treated sample of the Δ*Fgdbr1* strain showed a markedly higher proportion of intronic reads compared to the corresponding sample of the wild-type strain.

To validate the reliability of the RNA-seq data, we used qRT-PCR analysis to measure the transcript levels of two housekeeping genes and two selected ribosomal protein genes (RPGs) in samples of each strain with or without RNase R treatment. The transcript levels of these four selected genes were dramatically reduced in the RNase R-treated samples, suggesting that linear mRNA was nearly eliminated by RNase R treatment (Fig. 4c). Taken together, these results reveal that lariat RNA was successfully enriched through RNase R treatment and that a substantial quantity of intron lariats accumulated in the Δ*Fgdbr1* mutant.

### Transcript and intronic expression signatures in Δ*Fgdbr1*

**Fig. 5:**
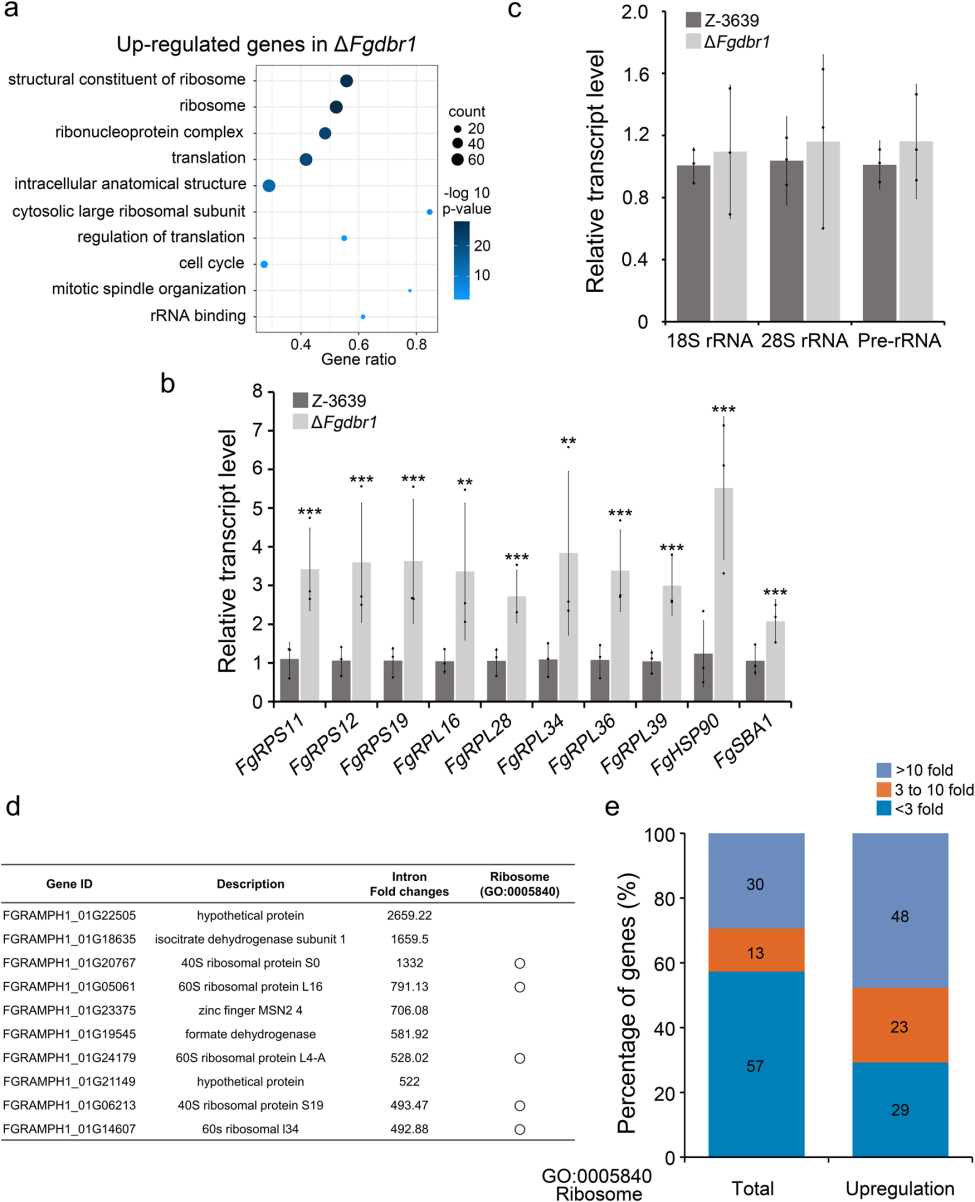
Transcript and intronic expression signatures in Δ*Fgdbr1* mutant. **a** Gene ontology (GO) enrichment analysis of the upregulated genes in the Δ*Fgdbr1* mutant. **b** Transcript levels of ten selected genes related to ribosome and translation in the *F. graminearum* strains. Transcript levels of selected genes were quantified by qRT-PCR. **c** Relative amount of ribosomal RNAs (rRNAs) in the wild-type and Δ*Fgdbr1* strains. 18 S, 28 S and pre-rRNA levels were quantified by qRT-PCR. Error bars represent standard deviations of three biological replicates. **p < 0.01; ***p < 0.001. **d** List of genes containing top 10 enriched intron lariat in Δ*Fgdbr1* mutant compared to wild type. **e** The proportion of genes with the intron lariat fold changes in three categories. Relative intron lariat levels of total genes or upregulated genes corresponding GO term ‘ribosome (GO:0005840) were analyzed based on RNA-set data of each strain.

Using RNA-seq data of RNase R-untreated samples containing intact mRNA, we identified differentially expressed genes (DEGs) displaying greater than two-fold changes in transcript levels of Δ*Fgdbr1* compared to the wild-type strain. The Δ*Fgdbr1* mutant exhibited 1161 upregulated and 1297 downregulated genes (Supplementary Data 1), which were subjected to gene ontology (GO) enrichment analysis. Upregulated genes in the Δ*Fgdbr1* mutant showed significant enrichment in functions related to the ribosome and translation, such as structural constituent of ribosome (molecular function), ribosome (cellular component), and translation (biological process) (Fig. 5a).

To verify the RNA-seq data, 10 upregulated genes related to the ribosome or translation were randomly selected and their transcript levels were measured through qRT-PCR. As shown in Fig. 5b, the transcript levels of these 10 genes increased by two- to three-fold in the Δ*Fgdbr1* strain. As many RPGs were upregulated, we investigated whether the numbers of ribosomal RNAs (rRNAs) differed between Δ*Fgdbr1* and the wild-type strain. We observed that the levels of 18 S rRNAs, 28 S rRNAs and pre-rRNAs in the wild-type and Δ*Fgdbr1* strains were similar, indicating that deletion of *FgDBR1* did not affect rRNA biogenesis (Fig. 5c). These results imply that upregulation of RPGs, not rRNA, leads to the imbalance between ribosomal components. This imbalance may cause impaired ribosome biogenesis and waste a large amount of cellular energy^20,21^.

As intronic read counts were markedly higher for the Δ*Fgdbr1* mutant than the wild type, we further analyzed the host genes harboring introns, of which abundance was increased in the Δ*Fgdbr1* mutant. Introns corresponding to 6677 genes were detected in our samples treated with RNase R. Compared to the wild type, introns of 643 (10%) and 623 (9%) host genes increased by more than ten-fold and by three- to ten-fold in the Δ*Fgdbr1* mutant, respectively. Notably, five host genes among the ten most highly accumulated introns were included in the GO term ‘ribosome’ (GO:0005840) (Fig. 5d). In addition, the transcript levels of these genes increased by two- to three-fold in the Δ*Fgdbr1* mutant compared with the wild type. We further investigated the number of host genes included in the GO term ‘ribosome’ for which the introns exhibited significant increases in the Δ*Fgdbr1* mutant. Of the total of 143 genes, 30% and 13% showed increased levels of intron lariats of more than ten-fold and of three- to ten-fold in the Δ*Fgdbr1* mutant, respectively (Fig. 5e and Supplementary Data 2). Furthermore, 65 of the 143 genes included in the GO term ‘ribosome’ were upregulated in the Δ*Fgdbr1* mutant by more than two fold, and in approximately 70% of those upregulated genes the abundance of intron lariats increased more than three fold.

**Fig. 6:**
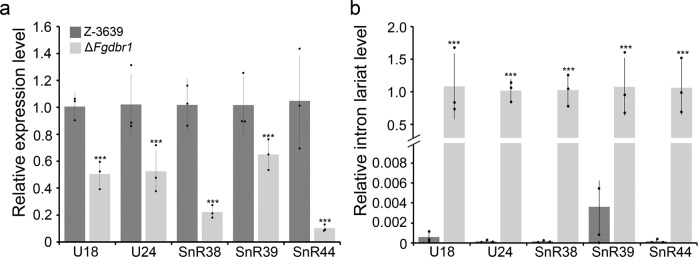
Effect of *FgDBR1* deletion on expression of snoRNA and intron lariat harboring snoRNA. **a** Relative expression level of intronic snoRNAs, U18, U24, SnR38, SnR39 and SnR44, in the wild-type and Δ*Fgdbr1* strains was quantified by qRT-PCR. **b** Relative expression level of intron lariats that harbor snoRNAs. Intron lariat level was measured by qRT-PCR in RNase R-treated samples. Error bars represent standard deviations from three biological replicates. ***p < 0.001).

In yeast, it has been known that debranching of intron lariat by Dbr1p is required for biogenesis of many intronic small nucleolar RNAs (snoRNAs)^22^. We evaluated the expressions of putative intronic snoRNAs in *F*. *graminearum* that were identified in the previous study^23^. Relative expressions of five putative intronic snoRNAs, U18, U24, SnR38, SnR39, and SnR44, were significantly decreased in the Δ*Fgdbr1* strain compared to those of the wild-type strain (Fig. 6a). In addition, intron lariats that possess the intronic snoRNAs were highly accumulated in the Δ*Fgdbr1* strain (Fig. 6b).

Taken together, these results suggest that overaccumulation of intron lariats due to the deletion of *FgDBR*1 may influence the expression of RPGs, but not rRNAs, and affects intronic snoRNA processing. Furthermore, we found a correlation between highly accumulated introns and the corresponding upregulated genes in Δ*Fgdbr1*. Based on these findings, we inferred that the correlation between highly accumulated introns and the corresponding upregulated RPGs may be due to the abundant expression of RPGs, the possibility of host gene regulation by intron lariats, or both.

### Validation of intron lariat RNAs in *F. graminearum* strains

**Fig. 7:**
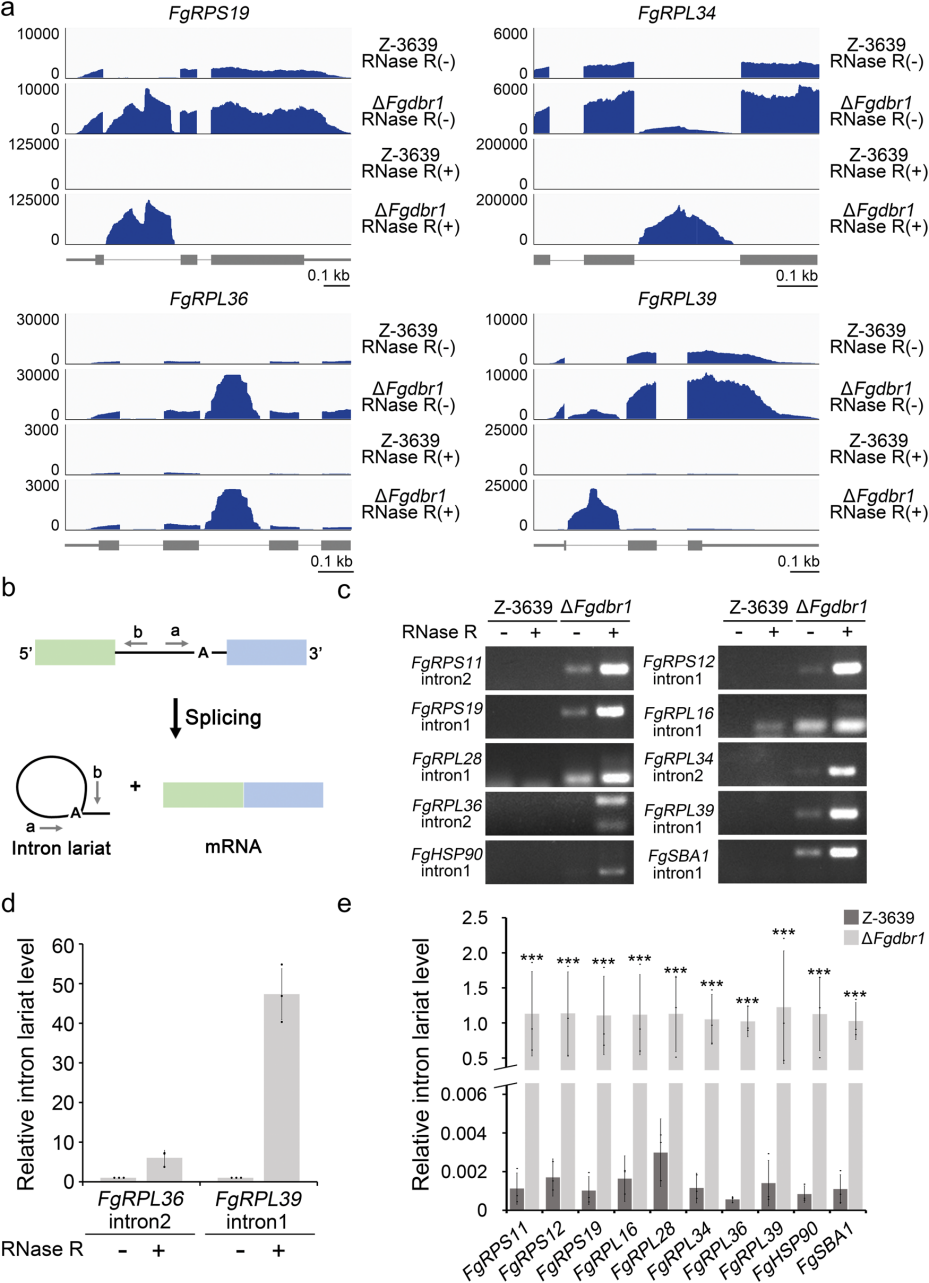
Identification of intron lariats in *F. graminearum*. **a** Genome browser images of four selected ribosomal protein genes. Aligned RNA-seq data generated from RNase R treated or untreated samples of *F. graminearum* strains were visualized using IGV. The *y*-axis represents the TPM. Thick box, coding sequence; thin box, untranslated region; line, intron. **b** Schematic representation of divergent primers used for amplifying intron lariat RNAs. Green and blue box, exon; line, intron; A, branch point. **c** Detection of intron lariat RNAs using reverse transcription PCR (RT-PCR) assays. RNA samples of each strain with or without RNase R treatment were amplified using divergent primers described in **a**. The images were obtained from 30 cycle of PCR using 2% agarose gels. **d** Verification of RNase R treatment. Relative intron lariat levels of Δ*Fgdbr1* samples with or without RNase R treatment were determined by qRT-PCR analysis. **e** Relative intron lariat levels in the wild-type and Δ*Fgdbr1* strains. Intron lariat RNA levels of RNase R treated samples were quantified by qRT-PCR. Error bars represent standard deviations of three biological replicates. ***p < 0.001.

We visualized the patterns of sequence alignment in intronic regions using Integrative Genomic Viewer (IGV). For samples treated with RNase R, very few exon-mapping RNA-seq reads were observed in the wild-type and Δ*Fgdbr1* strains compared to RNase R-untreated samples, as expected (Fig. 7a). Conversely, abundant intron-mapping reads were observed in the *Fgdbr1* mutant, but not in the wild-type strain. The abundance of intron-mapping reads in the RNase R-treated samples indicated that the intron lariats accumulated as stable circular forms at high levels in the deletion mutant.

To confirm intron lariat RNA identified from the RNA-seq, we designed divergent primer sets to amplify lariat RNA specifically^19^. In brief, the forward primer was designed to bind upstream of the branch point and the reverse primer was designed to bind downstream of the 5′ splice site (Fig. 7b). First, we performed reverse transcription PCR (RT-PCR) to detect intron lariats. In total, 10 intron lariats for previously selected genes were successfully amplified in the RNase R-treated sample of the Δ*Fgdbr1* mutant (Fig. 7c). In the sample from the Δ*Fgdbr1* mutant, most introns were detected regardless of RNase R treatment, whereas one intron was detected in the wild-type sample only with RNase R treatment. Furthermore, the intron lariat levels of two RPGs were quantified in the Δ*Fgdbr1* strain using divergent primers. As expected, the lariat RNAs were detectable regardless of RNase R treatment, and the levels of lariat introns were significantly higher in RNase R-treated samples (Fig. 7d). We also measured intron lariat levels in samples of each strain treated with RNase R using qRT-PCR analysis. Consistent with our RNA-seq results, high levels were observed in the Δ*Fgdbr1* sample, while in the wild-type sample intron lariats were generally undetectable (Fig. 7e).

### Effects of intron lariat overaccumulation on the sexual and asexual developmental stages

**Fig. 8:**
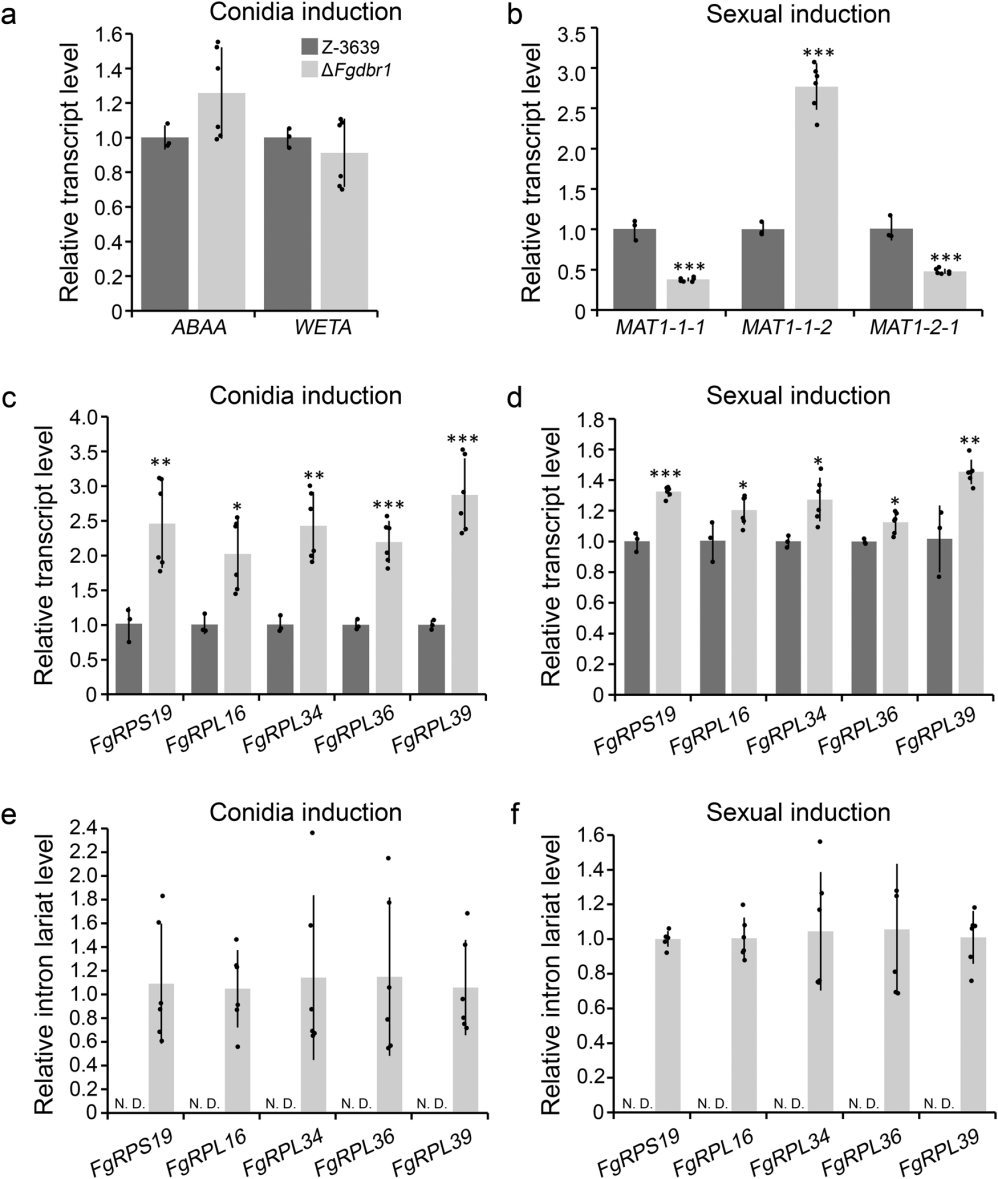
Effects of accumulated intron lariats on the transcript levels of conidiation related, mating-type genes, and ribosomal components. **a** Transcript levels of genes related to conidiation in the wild-type and Δ*Fgdbr1* strains. Transcript levels were quantified by qRT-PCR 12 h after conidia induction on YMA. **b** Transcript levels of mating-type genes in the wild-type and Δ*Fgdbr1* strains. Transcript levels were quantified by qRT-PCR 2 days after sexual induction. **c**, **d** Transcript levels of ribosomal protein genes in the wild-type and Δ*Fgdbr1* strains during conidia (**c**) and sexual induction (**d**). **e**, **f** Relative intron lariat levels in the wild-type and Δ*Fgdbr1* strains during asexual (**e**) and sexual development (**f**). Error bars represent standard deviations of biological replicates (*n* = 3 for wild type, *n* = 6 for Δ*Fgdbr1*). *p < 0.05; **p < 0.01; ***p < 0.001. N.D. not detected.

Our RNA-seq data showed that the expression levels of a considerable number of genes were changed as a result of the accumulated intron lariats. Therefore, we first investigated the transcript levels of conidiation related genes and mating-type (*MAT*) genes, to explore the underlying mechanisms of defective phenotypes in the deletion mutant during asexual and sexual development, respectively. We observed that the transcript levels of *ABAA* and *WETA*, the transcription factors involved in asexual development in *F*. *graminearum*^24,25^, were similar in both wild-type and Δ*Fgdbr1* strains 12 h after conidia induction (Fig. 8a). Interestingly, the expression levels of *MAT* genes were altered in the deletion mutant at the initial stage of sexual development (2 days after sexual induction), when compared to the wild type (Fig. 8b). While *MAT1-1-2* was upregulated, the expression levels of *MAT1-1-1* and *MAT1-2-1* were decreased in the Δ*Fgdbr1* mutant, indicating that deletion of *FgDBR1* affects the expression of *MAT* genes at the initial stage of sexual development in *F. graminearum*.

Furthermore, we measured the transcript levels of 5 RPGs after asexual and sexual induction, as many RPGs were upregulated in the deletion mutant grown in liquid CM. Consistent with the results in vegetative growth, Δ*Fgdbr1* exhibited increased expression levels of RPGs after conidia induction, while showed slightly increased levels after sexual induction, compared with the wild-type strain (Fig. 8c, d). We also investigated intron lariat levels of 5 RPGs in each strain using qRT-PCR analysis. As with our RNA-seq results, high levels of lariats were observed in the Δ*Fgdbr1*, while intron lariats were generally undetectable in the wild type (Fig. 8e, f). Together, the results demonstrate that overaccumulation of intron lariats in the deletion mutant led to upregulation of RPGs after conidia and sexual development induction, which lead to the imbalance between ribosomal components in both asexual and sexual developmental stages, like vegetative growth.

### Deletion of *FgDBR1* affects protein synthesis in *F. graminearum*

**Fig. 9:**
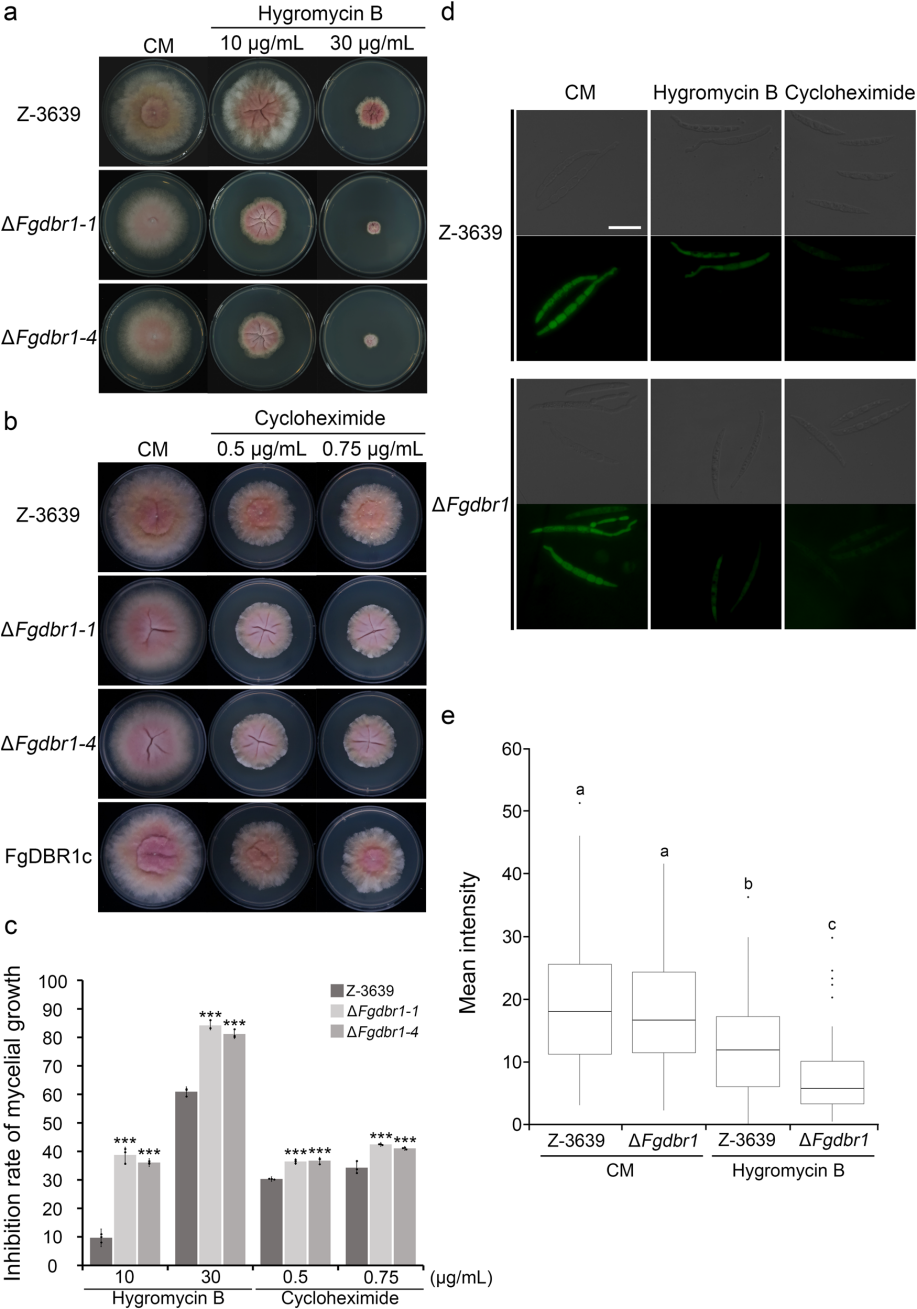
Reduced protein translation rate in the Δ*Fgdbr1* strains. **a** Mycelial growth of *F. graminearum* strains on CM and CM supplemented with hygromycin B. **b** Mycelial growth of *F. graminearum* strains on CM and CM supplemented with cycloheximide. The photographs were taken 5 days after inoculation. **c** Inhibition rate of mycelial growth in response to protein synthesis inhibitors. The percentage of mycelial growth inhibition was examined 5 days after inoculation on CM with protein synthesis inhibitors. Error bars represent standard deviations of three biological replicates. ***p < 0.001. **d** Visualization of newly synthesized proteins in germinating conidia of *F. graminearum* strains. Click-iT protein synthesis assay kit was used to visualize protein translation. Conidia of each strain were inoculated in liquid CM with or without translation inhibitor and processed for click reaction. The newly synthesized proteins were examined under a fluorescence microscope using the filter L5. Scale bar = 25 μm. **e** Quantification of the mean fluorescent intensity of each sample. The mean GFP fluorescence intensity in a transverse section of individual conidium, indicating newly synthesized proteins, was measured using LAS x software. A total of 100 conidia was observed in each sample. Lower and upper whiskers represent 1.5 times of the lower and upper interquartile ranges (IQR), respectively, and median values were indicated by solid black lines. Same letters mean there is no significant difference between strains, different letters indicate statistically significant differences with *p* < 0.01.

Based on our transcriptome analysis, we hypothesized that upregulation of numerous RPGs in the Δ*Fgdbr1* mutant leads to impaired ribosome biogenesis and consequent protein translation. Therefore, to investigate the relationship between protein translation efficiency and the role of *FgDBR1*, we first tested the sensitivity of the wild-type and Δ*Fgdbr1* strains to the translation inhibitors hygromycin B and cycloheximide. The Δ*Fgdbr1* mutants exhibited markedly increased sensitivity to translation inhibitors compared with the wild-type strain, and inhibition rates were higher in CM supplemented with hygromycin B than in CM supplemented with cycloheximide (Fig. 9a–c). Therefore, to determine whether protein translation was affected by deletion of *FgDBR1*, we measured overall protein synthesis in the wild-type and Δ*Fgdbr1* strains using a fluorescence-based translation assay. In this assay, O-propargyl-puromycin (OPP) is incorporated into newly translated proteins and fluorescently labeled with a green fluorescent azide dye, Alexa Fluor®.

Before the fluorescence-based translation assay, the conidial germination of each strain was examined in liquid CM and in CM supplemented with protein-synthesis inhibitors (10 or 30 ppm hygromycin B or 2.5 or 5 μM cycloheximide) to determine the appropriate experimental conditions. After incubation for 6 h in liquid culture, conidial germination of Δ*Fgdbr1* was significantly reduced in CM with hygromycin B compared to the wild type, while no significant difference was found between the two strains in CM with cycloheximide. Specifically, approximately 50% of conidia in the wild-type strain germinated and produced germ tubes, while fewer than 10% of conidia germinated in the deletion mutant, and they produced very short germ tubes. Thus, protein translation in germinating conidia was examined in liquid CM with or without 30 ppm hygromycin B; CM supplemented with 100 μM cycloheximide was used as a control. When incubated in liquid CM, the conidia of both strains produced strong green fluorescence signals of similar intensity, whereas when treated with 100 μM cycloheximide conidia did not exhibit fluorescence (Fig. 9d, e). When conidia were incubated in CM with hygromycin B, the Δ*Fgdbr1* mutant exhibited significantly lower fluorescence signal intensity compared to incubation in CM without hygromycin B, while the wild-type strain showed slightly lower intensity, indicating that overall protein translation was severely inhibited in the Δ*Fgdbr1* mutant. Taken together, these results suggest that excessive accumulation of intron lariats may directly or indirectly regulate the expression of RPGs, resulting in a reduced rate of protein synthesis.

## Discussion

Pre-mRNA splicing is an essential process for proper gene expression in eukaryotic organisms. The splicing process is mediated by a large RNA-protein complex, the spliceosome, which releases intron lariats with 2′-5′ phosphodiester bonds^26^. The removed introns are linearized by the RNA lariat debranching enzyme Dbr1 and then rapidly processed for degradation^4^ or further RNA biogenesis by several exoribonucleases^9,27^. Because eukaryotic genes generally contain introns, pre-mRNA splicing and the intron turnover processes are highly conserved among species as diverse as yeast and humans. Therefore, the molecular functions of Dbr1 have been studied in model organisms, including yeast, *Arabidopsis*, and humans. Specifically, several studies focused on human diseases have demonstrated that RNA metabolism controlled by Dbr1 affects cancer development and viral infection^28,29^. However, the detailed function of Dbr1 in filamentous pathogenic fungi, including *F. graminearum*, remains unclear. In this study, we characterized *FgDBR1*, which can complement the phenotypic defect in the yeast *DBR1* deletion mutant. Through transcriptome analysis, we revealed that deletion of *FgDBR1* resulted in genome-wide accumulation of intron lariat RNAs. These results support the intron lariat debranching activity of FgDbr1.

Although Dbr1 is evolutionarily well conserved, the role of Dbr1 is more important in organisms that have a substantial number of introns than organisms with a small number of introns in their genomes. The phenotypes resulting from *DBR1* deletion differ among species. While the deletion mutants of yeast species were viable, deletion of *DBR1* resulted in severe growth defects in *S. pombe* but not in *S. cerevisiae*, which have genomes with 40% and 2.5% intron-containing genes, respectively^6^. Furthermore, CRISPR/Cas9 mediated knockout of *DBR1* demonstrated that Dbr1 protein is essential for cell survival in humans, as the human genome includes mostly intron-containing genes (over 92%)^30,31^. The genome of *F. graminearum* contains 77% intron-bearing genes^32^. While deletion of the *DBR1* gene in *S. cerevisiae* did not affect growth, deletion of *FgDBR1* resulted in multiple defects, including impaired hyphal growth, conidiation, sexual reproduction, and virulence, indicating that the function of Dbr1 is important for the development and pathogenesis of filamentous fungi.

Previous studies have revealed that overaccumulation of intron lariats affects gene-expression regulation at various stages. In *Arabidopsis*, increased abundance of lariat RNAs inhibited genome-wide microRNA (miRNA) biogenesis; intron lariats may be recognized by the dicing complex and the association between lariat RNA and the dicing complex may lead to reduced binding of the primary miRNA to the dicing complex^16^. Reduced expression of hDBR1 resulted in accumulation of intron lariats, causing the splicing complexes to become trapped by intron lariats. Thus, reduced active splicing complex abundance led to splicing defects in humans^24^. Also, increased levels of lariat RNA reduced the overall efficiency of mRNA splicing in *S. pombe*^15,33^. Therefore, we measured the intron splicing ratios of four selected RPGs via qRT-PCR, as described previously^34^. The splicing ratios of the tested genes in Δ*Fgdbr1* did not differ significantly from those in the wild-type strain, suggesting that excessive accumulation of intron lariats does not seriously affect the splicing ratio at the genome-wide level in *F. graminearum* (Supplementary Fig. 6). Furthermore, the *DBR1* deletion mutant of *S. pombe* exhibited an increased polysome-to-monosome ratio compared to the wild type. This result implies that ribosomal occupancy of transcripts is elevated due to a reduced translation rate at the elongational level in the absence of *DBR1* in *S. pombe*. Likewise, combination of transcriptome analysis and a fluorescence-based protein synthesis assay revealed that deletion of *FgDBR1* resulted in upregulation of numerous RPGs and consequent reduced translation capacity in *F. graminearum*. Therefore, we conclude that compromised translation in the Δ*Fgdbr1* mutant causes severe phenotypic defects in virulence, conidiation, sexual reproduction, and stress sensitivity.

Because 60% of total transcription is devoted to rRNA production and 50% of RNA polymerase II transcription is devoted to RPGs, the process of ribosome biogenesis accounts for a large proportion of cellular energy demand^21^. Therefore, ribosome biogenesis is tightly regulated, and ribosomal components are synthesized and assembled in a coordinated manner^35^. An imbalance between ribosomal protein (RP) and rRNA production leads to nucleolar stress and impairment of the cell cycle in both yeast and mammalian cells^20,36^. Our transcriptome analysis revealed that numerous ribosome- and translation-related genes and their corresponding introns were significantly upregulated in Δ*Fgdbr1* compared to the wild-type strain. While many RPGs were upregulated in the deletion mutant, transcript levels of rRNAs were not elevated, indicating an excessive number of RPs relative to rRNAs. This difference may lead to impaired ribosome biogenesis and wasted substantial amounts of cellular energy and materials, resulting in decreased translation in the Δ*Fgdbr1* mutant. In addition, the decreased level of protein synthesis in the deletion mutant may positively regulate the expression of RPGs. Moreover, it is reasonable to propose that the effects of reduced protein translation may be greater in stages when nutrients are limited and a significant amount of energy is needed, such as during asexual and sexual reproduction and pathogenesis, than in nutrient-replete stages such as vegetative growth.

Understanding that introns play a wide range of biological functions is increasing^37^. In human cell lines, introns that are not completely degraded and accumulate in circular forms regulate local gene expression^38^. Regulation of growth by excised linear introns was observed in yeast, suggesting that introns can have biological functions^39^. Additionally, introns may be processed to non-coding RNA, such as miRNA, snoRNA, and various types of long non-coding RNA (lncRNA). In yeast, previous research showed that some snoRNAs derived from introns, and their production levels, were dependent on the Dbr1 protein^22^. In the present study, we observed that the read counts of some intron lariats were relatively high in the wild-type strain. This result indicates that some intron lariats of *F. graminearum* naturally escape debranching by Dbr1 and accumulate in a circular form, as observed in plant and vertebrate cells, and that introns of this plant pathogenic fungus may play specific roles in cellular metabolism^16,40^. Additionally, within the GO term “ribosome”, a significant correlation was found between upregulated genes and genes with higher accumulation of introns in the Δ*Fgdbr1* mutant compared to the wild-type strain. We assume that these correlations are because RPGs generally have much higher expression levels compared to other genes, and intron lariats from RPGs may directly or indirectly regulate the expression of their host genes. Therefore, the regulatory functions of these intron lariats need to be investigated in future research.

In summary, the Δ*Fgdbr1* mutant exhibited various defects, especially in conidiation, sexual reproduction, and plant infection. Through various genetic approaches, we revealed that a compromised intron turnover process due to the absence of *FgDBR1* resulted in decreased translation, not only transcription level, in *F. graminearum*. Therefore, reduced overall protein synthesis contributes to the severe defects in virulence and other developmental processes, including asexual and sexual development, observed in Δ*Fgdbr1*. Our results suggest that RNA homeostasis via rapid turnover of cellular RNA is important for the development and virulence of the plant pathogenic fungus *F. graminearum*.

## Methods

### Fungal strains and media

The *F. graminearum* wild-type strain Z-3639^41^ and mutants derived from this strain were stored as mycelial suspensions in 20% glycerol at −80 °C. All strains used in this study are listed in Supplementary Data 3. Culture media were prepared following the *Fusarium* laboratory manual^42^. Conidia of all strains were induced either in carboxymethyl cellulose (CMC) medium or on yeast malt agar (YMA)^43,44^.

To determine the sensitivity of *F. graminearum* to various stress conditions, a mycelial plug from actively growing culture was placed on a CM plate supplemented with various stress reagents^45^. In addition, the DNA-damaging agents used in this study were hydroxyurea (HU) and methyl methanesulfonate (MMS). The tested conditions included oxidative stress (5 mM hydrogen peroxide and 40 μM menadione), osmotic stress (1 M NaCl, 1 M KCl, 1.5 M sorbitol and 4 mM FeSO_4_), cell-wall and membrane stress (100 mg/L sodium dodecyl sulfate and 60 mg/L Congo Red), inhibition of the mitogen-activated protein kinase pathway (0.02 mg/L fungicide fludioxonil), inhibition of DNA synthesis (7 mg/L fungicide iprodione), inhibition of meiosis (0.65 mg/L fungicide benomyl), azole fungicides (0.025 mg/L tebuconazole), pH stress (pH **=** 4 and pH = 11), and DNA damage stress (10 mM HU and 0.1 μL/mL MMS).

### Nucleic acid manipulations and genetic modifications

Fungal genomic DNA was extracted from freeze-dried mycelia powder according to the *Fusarium* laboratory manual^42^. Total RNA was isolated from mycelia ground in liquid nitrogen using an Easy-Spin Total RNA Extraction Kit (Intron Biotech, Seongnam, Republic of Korea). Restriction endonuclease digestion and agarose gel electrophoresis were performed using standard protocols^46^. A North2South Biotin Random Prime Labeling Kit and a North2South Chemiluminescent Hybridization and Detection Kit (Thermo Scientific, Waltham, MA, USA) were used for Southern blot hybridization. DNA sequencing was performed by Macrogen (Seoul, Korea) and the primers used in this study (Supplementary Table 1) were synthesized at an oligonucleotide synthesis facility (Bioneer, Daejeon, Korea).

The fusion PCR products required for targeted gene deletion were generated via the double-joint (DJ) PCR strategy^47^. The geneticin resistance cassette (*GEN*) was amplified from pII99^48^, and the 5′ and 3′ flanking regions of *FgDBR1* were amplified from genomic DNA of the wild-type strain. The three resultant amplicons were fused through a second round of DJ PCR. The final constructs were generated in a third round of PCR using nested primers and transformed into wild-type protoplasts, using PEG-mediated transformation^49^. For complementation of the Δ*Fgdbr1* strain, the *FgDBR1-GFP* fusion construct was generated via the yeast gap repair approach^50^. The ORF of *FgDBR1* and 1 kb of its upstream promoter region were amplified and co-transformed with the XhoI-digested vector pDL2^51^ into the yeast strain PJ69-4A. Subsequently, the *FgDBR1-GFP* fusion vector was rescued from the yeast transformants and transformed into *Escherichia coli* DH10B. After verification through sequencing, the resulting vector was transformed into the Δ*Fgdbr1* mutant.

### Conidium production and morphology

After incubating a cultured plug of CM in 5 mL CMC medium for 5 days at 25 °C on a rotary shaker (200 rpm), conidium production was evaluated by counting the numbers of conidia using a hemocytometer (Superior, Marienfeld, Germany). To observe conidium morphology, conidia induced on YMA were harvested and differential interference contrast (DIC) images were obtained using a DM6 B microscope (Leica Microsystems, Wetzlar, Germany).

### Sexual crosses

To induce sexual reproduction, the wild-type and mutant strains were incubated on carrot agar plates at 25 °C for 5 days^42^. Mycelia grown on carrot agar were mock fertilized with sterile 2.5% Tween 60 solution for self-fertilization. For outcrosses, the heterothallic female strain was fertilized with 1 mL of conidial suspension from a male strain (10^6^ conidia/mL) at 5 days after inoculation on carrot agar. All fertilized cultures were incubated at 25 °C for 7–10 days under near-ultraviolet light (wavelength, 365 nm; HKiv Import and Export Co., Ltd., Xiamen, China).

### Lariat RNA enrichment and RNA-seq analysis

Conidial suspensions of the wild-type and Δ*Fgdbr1* strains were inoculated in liquid CM (1 × 10^5^ conidia/mL) and mycelia were harvested after 24 h of incubation on a rotary shaker (200 rpm). Total RNA was extracted from each mycelial sample and RNA quality was assessed on the Agilent 2100 Bioanalyzer system (Agilent Technologies, Santa Clara, CA, USA). Total RNA from each sample was used for RNA-seq library construction using an Illumina TruSeq Stranded mRNA Library Prep Kit (Illumina, San Diego, CA, USA) following the manufacturer’s protocol. To remove linear RNA, 10 μg total RNA was treated with RNase R (BioVision, Milpitas, CA, USA) at 37 °C and purified using an RNeasy MinElute Cleanup Kit (Qiagen, Hilden, Germany). The purified RNA was subjected to RNA-seq library preparation using a TruSeq Stranded Total RNA with Ribo-Zero H/M/R Sample Prep Kit following the manufacturer’s instructions. Sequencing was performed on an Illumina NovaSeq6000 system. Total RNA samples without RNase R treatment were sequenced as controls and were used for comparison of global transcriptome analysis between the wild type and Δ*Fgdbr1*. The quality of raw reads was evaluated using the FastQC tool (http://www.bioinformatics.babraham.ac.uk/projects/fastqc/) and the FASTX-Toolkit filtered out low-quality reads (50% of sequence ≥28 quality score) (http://hannonlab.cshl.edu/fastx_toolkit/). Alignment was performed with the STAR program^52^ using the *F. graminearum* genome^32^, and the featureCounts function was used to compute the counts for each gene^53^. Differential expression analysis of genes was performed with DESeq2^54^ and differentially expressed genes (*P* < 0.05 and fold change of ≥2) were subjected to GO analysis using GOseq^55^. Raw data from RNA-seq were deposited in the National Center for Biotechnology Information Sequence Read Archive (BioProject PRJNA792684).

### Reverse transcription PCR and quantitative real-time PCR

Both total RNA and RNase R-treated RNA were subjected to first-strand cDNA synthesis using the SuperScript III First-Strand Synthesis System (Invitrogen, Carlsbad, CA, USA) with random hexamers or Oligo(dT)_20_ primers. qRT-PCR was performed using iTaq Universal SYBR Green Supermix (Bio-Rad, Hercules, CA, USA) with the CFX Real-Time PCR System (Bio-Rad), and data were collected using the Bio-Rad CFX Maestro Software. The endogenous housekeeping gene ubiquitin C-terminal hydrolase (*UBH1*) and elongation factor 1α (*EF1α*) was used as an internal control for normalization. Relative expression levels were calculated through the 2^-ΔΔCT^ method^56^. For lariat RNA detection, divergent primer sets, which can amplify lariat RNA across the branch site, were designed to face away from each other on the linear RNA and span the back-splice junction sequence of lariat RNAs^19^. For detection of snoRNA, small RNA cDNA (srcDNA) library was prepared from polyadenylated RNA^57^. Briefly, 5 μg of Total RNA was polyadenylated using *E*. *coli* poly(A) polymerase (Enzynomics, Daejeon, Korea) and purified using RNA Clean & Concentrator -5 kit (Zymo Research, Irvine, CA, USA). The poly(A)-tailed RNA was reverse transcribed using SuperScript III First-Strand Synthesis System with miRTQ primer (Supplementary Table 1). qRT-PCR of each snoRNA was performed using snoRNA-specific primer and a universal reverse primer, RTQ-UNIr. PCR assays were repeated three times with three biological replicates.

### Virulence assay and mycotoxin analysis

To assess virulence, the point inoculation method was employed using the wheat cultivar Eunpamil. Conidial suspension (10 μl, 1 × 10^5^ conidia/mL) obtained from each strain was injected into the center spikelet of a wheat head at early anthesis^14^. Inoculated plants were grown in a greenhouse after 3 days of incubation in a humidity chamber. Infected wheat heads were imaged and the numbers of spikelets with typical head-blight symptoms were counted at 21 days post-inoculation. Ten replicate inoculations in each of two independent mutant strains were used in the experiment.

To measure trichothecene production, conidial suspension (1 × 10^4^ conidia/mL) was inoculated in defined medium containing 5 mM agmatine (MMA) and incubated for 7 days at 25 °C under stationary growth conditions^58^. MMA cultures were extracted with ethyl acetate-methanol mixture (4:1, v/v) and the resulting extracts were dried. The residue was resuspended in the mobile phase (10% acetonitrile solution) and analyzed using a Shimadzu UFCL system with an RF-10A XL fluorescence detector (Shimadzu, Kyoto, Japan). The total trichothecene concentration was calculated based on the biomass produced by each strain in MMA.

### Microscopic observation

Microscopic observation was performed with a DM6 B microscope using an L5 fluorescence filter (excitation 480/40 nm; emission 527/30 nm) for GFP and an N2.1 filter (excitation 515–60 nm; emission 590 nm) for red fluorescent protein. To observe the spread of mycelia on wheat heads, wheat heads were inoculated with the cytosolic GFP-expressing strains and harvested at 6 days post-inoculation. Freehand longitudinal sections across the center of infected spikelets were prepared using a clean scalpel^59^. Sectioned wheat heads were imaged under reflected light and GFP fluorescence settings (470-nm-excitation and 525-nm-emission wavelength filters) using a SteREO Lumar V12 microscope (Carl Zeiss, Oberkochen, Germany).

### Heterologous expression in yeast

The *S. cerevisiae* wild-type strain (BY4741) and Δ*Scdbr1* strain (YKL149C) were obtained from EUROSCARF (Frankfurt, Germany). The full-length cDNA sequence of *FgDBR1* was amplified, digested with EcoRI and XbaI, and inserted into the yeast expression vector pYES2 (Invitrogen, Carlsbad, CA, USA). The cloned vector pYES2-FgDBR1 was transformed into the Δ*Scdbr1* strain using an Alkali-Cation Yeast Transformation Kit (MP Biomedicals, Santa Ana, CA, USA). Additionally, the empty pYES2 vector was introduced into the wild-type and mutant yeast strains. Yeast transformants were selected on synthetic-defined medium lacking uracil (SD-Ura) and further confirmed through PCR amplification of *FgDBR1*. For complementation assays, each transformant was grown in SD-Ura medium supplemented with 2% galactose overnight at 30 °C in a rotary shaker (200 rpm). Yeast cells were re-inoculated in fresh SD-Ura supplemented with 2% galactose until the optical density at 600 nm reached 0.6–0.8. The cells were harvested through centrifugation, total RNA was isolated, and relative intron lariat levels were analyzed using qRT-PCR. The endogenous *S. cerevisiae* housekeeping gene actin (*ACT1*) was used for normalization.

### Fluorescent protein synthesis assay

To measure newly synthesized proteins in fungal cells, a Click-iT™ Plus OPP Alexa Fluor™ 488 Protein Synthesis Assay Kit (Thermo Scientific) was used according to the manufacturer’s instructions. Briefly, conidia induced on YMA were harvested and inoculated in liquid CM (1 × 10^6^ conidia/mL). After 1 h of incubation on a rotary shaker, cells were washed with phosphate-buffered saline (PBS). Then, the cells were re-inoculated in liquid CM containing OPP with or without translation inhibitors (cycloheximide or hygromycin B) and incubated for 1 h on a rotary shaker. The cells were fixed using 3.7% formaldehyde solution and permeabilized with 0.5% Triton X-100 in PBS. Reaction cocktail containing Alexa Fluor® dye with the azide moiety was prepared for the click reaction and added to the cells. Samples were incubated for 30 min at room temperature in the dark and then washed with rinse buffer. The newly synthesized proteins were examined with a DM6 B microscope (Leica Microsystems) using the L5 filter set with a consistent exposure time. After the images had been captured, mean GFP fluorescence intensity in the transverse section of an individual conidium was quantified using LAS X software (Leica Microsystems). In total, 100 conidia from each sample were examined.

### Statistics and reproducibility

All experiments were repeated at least three times with three replicates each, unless indicated otherwise. Error bars indicate standard deviations. Statistical analyses were performed using R v3.5.3. Comparisons between multiple groups were performed with one-way ANOVA followed by Tukey’s multiple-comparison test. Two-tailed unpaired Student’s *t* test was used for comparisons of two groups. *P*-values less than 0.05 were considered statistically significant (*P <  0.05, **P < 0.01, ***P <  0.001).

### Reporting summary

Further information on research design is available in the Nature Research Reporting Summary linked to this article.

## Supplementary information

Supplementary information

Description of Additional Supplementary Data

Supplementary Data 1

Supplementary Data 2

Suppelmentary Data 3

Supplementary Data 4

Reporting Summary

## Data Availability

RNA-sequencing data were deposited in the NCBI database, and can be found under the BioProject accession number PRJNA792684. Source data underlying figures in the main text are available in Supplementary Data 4.
