# Supplementary material for: Intron turnover is essential to the development and pathogenicity of the plant pathogenic fungus Fusarium graminearum
Source: Commun Biol. 2022 Oct 26;5:1129. doi: 10.1038/s42003-022-04111-3 (PMC9606315; doi:10.1038/s42003-022-04111-3)
Supplement: Supplementary file 1 — Supplementary information [file 42003_2022_4111_MOESM1_ESM.pdf]

Supplementary information

Intron turnover is essential to the development and pathogenicity of the plant pathogenic fungus *Fusarium graminearum*

Yejin Choi[1], Hyun-Hee Lee[2], Jiyeun Park[1], Sieun Kim[1], Soyoung Choi[1], Heeji Moon[1], Jiyoung Shin[3], Jung-Eun Kim[3], Gyung Ja Choi[4], Young-Su Seo[2], and Hokyoung Son[1, 3*]

[1]Department of Agricultural Biotechnology, Seoul National University, Seoul 08826, Republic of Korea

[2] Department of Integrated Biological Science, Pusan National University, Busan 46247, Republic of Korea

[3] Research Institute of Agriculture and Life Sciences, Seoul National University, Seoul 08826, Republic of Korea

[4] Therapeutic & Biotechnology Division, Center for Eco-friendly New Materials, Korea Research Institute of Chemical Technology, Daejeon 34114, Republic of Korea

Correspondence

Hokyoung Son, Department of Agricultural Biotechnology, Seoul National University, Seoul, Republic of Korea.

Email: hogongi7@snu.ac.kr

Supplementary Table 1. Primers used in this study.

Primer	Sequence (5′ → 3′)	Description
DBR1-5F	AGCGGGCTCTACAGGCTAACAG	Forward and reverse primers for amplification of 5' flanking region of *FgDBR1* with tail for the geneticin resistance gene cassette fusion
DBR1-5R	gcacaggtacacttgtttagagACGAAACATGGATACAAGGGAAAC	
DBR1-3F	ccttcaatatcatcttctgtcgCCTTGCAAGAATGGATGACGATA	Forward and reverse primers for amplification of 3' flanking region of *FgDBR1* with tail for the geneticin resistance gene cassette fusion
DBR1-3R	TGACAGCTAACCACTCGTTCTCGT	
DBR1-5N	GGTCTATGTTGCCAACACGCTGT	Forward and reverse nest primers for third fusion PCR for amplification of *FgDBR1* deletion construct
DBR1-3N	GTCACCTCAGCATACTGTCGCAG	
Gen-for	CGACAGAAGATGATATTGAAGG	Forward and reverse primers for amplification of the geneticin cassette from the pII99 vector
Gen-rev	CTCTAAACAAGTGTACCTGTG	
DBR1-GFP F	tatagggcgaattgggtactcaaattggttAGTACTTACTACAGGCCATTGATACAGATG	Forward and reverse primers for amplification of *FgDBR1* open reading frame for GFP fusion protein construction under native promoter
DBR1-GFP R	cccggtgaacagctcctcgcccttgctcacCCAGCGGCCTCCACGACC	
DBR1-cloning F	gcatgagaattcCTACCACAAAATGACTACCAACGC	Forward and reverse primers for amplification of *FgDBR*1 cDNA for pYES2 cloning
DBR1-cloning R	gcatgatctagaTTACCAGCGGCCTCCACG	
DBR1-seq-F	AGTAACCTGGCCCCACAAACCT	Forward and reverse primers for pYES2-FgDBR1 plasmid identification
DBR1-seq-R	CTTCGAGCGTCCCAAAACCTT	
ScACT1m-RT-F	CTCCTCGTGCTGTCTTCCCATCT	For real-time PCR of *S. cerevisiae ACT1*
ScACT1m-RT-R	ATCTTTTCCATATCGTCCCAGTTG	
ScACT1-I-RT-F	CCACGATATTATTGGAATAAATAGGGGC	For real-time PCR of *S. cerevisiae ACT1* intron
ScACT1-I-RT-R	TGGGACCGTGCAATTCTTCTTACA	
ScRPL28-RT-F	GGTTGTAGAGAGCGCAATTATGAAAAA	For real-time PCR of *S. cerevisiae RPL28* intron
ScRPL28-RT-R	ATACACACGACATATTGGTTGCACAAC	
ScRPS17A-RT-F	CGCCATGAACTGCCTCTACACATT	For real-time PCR of *S. cerevisiae RPS17A* intron

ScRPS17A-RT-R	CCACTTTTTCGGTATCGCAGTTCC	
UBH-RT-F	GTTCTCGAGGCCAGCAAAAAGTCA	For real-time PCR of *UBH1*
UBH-RT-R	CGAATCGCCGTTAGGGGTGTCTG	
EF1α-RF-F	GGCTTTCACCGACTACCCTCCTCT	For real-time PCR of *EF1α*
EF1α-RF-R	ACTTCTCGACGGCCTTGATGACAC	
RPS11-RT-F	CTTTCCAGAAGCAGCCTCACATCTT	For real-time PCR of *RPS11*
RPS11-RT-R	CTGCCCTCAATGGCGGTCTT	
RPS12-RT-F	CCCAAGGGCCAGATGTCCATT	For real-time PCR of *RPS12*
RPS12-RT-R	CTCACAGTTCTCGTTCAGGACACACA	
RPS19-RT-F	ACGTCTACCTCCGCAAGACTGTCG	For real-time PCR of *RPS19*
RPS19-RT-R	GCTCAAGGACACCGATCTTCTCAA	
RPL16-RT-F	CCAAGCAGCTCCTTAGTGGTCAGAA	For real-time PCR of *RPL16*
RPL16-RT-R	GTGGGGTTGTATCGGGTGATCTTT	
RPL28-RT-F	TTACTTCGGAAAGGTTGGTATGCG	For real-time PCR of *RPL28*
RPL28-RT-R	ACGTAGGCGTCACGGGTCTCTT	
RPL34-RT-F	TCACATCAAGAAGCGAGGCACTG	For real-time PCR of *RPL34*
RPL34-RT-R	ACCGCATCGAGAACCACCGTAA	
RPL36-RT-F	AACCAAGGGTCACCTGAGCAAGC	For real-time PCR of *RPL36*
RPL36-RT-R	CTTACGGGCACGCTTGTCCTT	
RPL39-RT-F	AAGTCTTTCCGAACCAAGCAGAAGC	For real-time PCR of *RPL39*
RPL39-RT-R	AGTGCCTTCGCTTGGCGTTGTA	
HSP90-RT-F	ATGATCGGTCAGTTCGGTGTCG	For real-time PCR of *HSP90*
HSP90-RT-R	TGATCTTGCTCTCGTTCAGGTAGTCG	
SBA1-RT-F	GAACTTCATCTACCTCACCATCTCCGT	For real-time PCR of *SBA1*
SBA1-RT-R	TGGAAGTGTGGTTGATCTTGCTCTC	
MAT1-1-1-RT-F	CTGGAAGAACTGGGCATCGTAA	For real-time PCR of *MAT1-1-1*

MAT1-1-1-RT-R	GATATTCTTGTGGCTGGCTACTTT	
MAT1-1-2-RT-F	CTATGCCTGCATCCTGAAGTCCA	For real-time PCR of *MAT1-1-2*
MAT1-1-2-RT-R	CATTATCGAAACCAAGGTCAAGTAGCA	
MAT1-2-1-RT-F	TGGCAGACGACATTAAGGAGGAGCAC	For real-time PCR of *MAT1-2-1*
MAT1-2-1-RT-R	TGAGCAGCGACAGCAGCAGCAAGAA	
abaA-RT-F	ACGCAAGCAAGTCTCAAGTCATA	For real-time PCR of *abaA*
abaA-RT-R	TGTTCCTCCTCGTCATAGTAATCA	
wetA-RT-F	GTTCCAGGTACTCCCACTGCCAT	For real-time PCR of *wetA*
wetA-RT-R	ACGTTCTCGTCGCGCTTTGGT	
RPS11-I-RT-F	CGACACACGAAATCACGACCATG	For real-time PCR of *RPS11* intron
RPS11-I-RT-R	CGATTTCGTTCCTTGTCTTCCAAA	
RPS12-I-RT-F	GGGGGAAGAGCAGCAAGAAAATAAT	For real-time PCR of *RPS12* intron
RPS12-I-RT-R	CTGACGTCGCGAATTGGTTCTC	
RPS19-I-RT-F	TGAAAACGCTGTGTCGACTGCTATG	For real-time PCR of *RPS19* intron
RPS19-I-RT-R	TGTTGCCAAGTCGTCGAGTAGAGAT	
RPL16-I-RT-F	AGCGCCTGCACTTCTTGTTGAC	For real-time PCR of *RPL16* intron
RPL16-I-RT-R	ATCCCAAAACGTCTCCAATCCCAT	
RPL28-I-RT-F	CACGACGAATCCCGACGAAATC	For real-time PCR of *RPL28* intron
RPL28-I-RT-R	CTCGCAGAAGCAAGAGTATAGCAAAAA	
RPL34-I-RT-F	GATTCGTGTCGTCTTGCTCCATCA	For real-time PCR of *RPL34* intron
RPL34-I-RT-R	CATGGTGGTGTCTCAATCGTTCG	
RPL36-I-RT-F	CTGGTGCGGCTATCTGTGTCTGTA	For real-time PCR of *RPL36* intron
RPL36-I-RT-R	GGTTCGTCCATCGGGTATCCTCT	
RPL39-I-RT-F	CCGTCGATAATGAGGTTGATGTTG	For real-time PCR of *RPL39* intron
RPL39-I-RT-R	GACATCGTCGATAATGAAGATTGCG	
HSP90-I-RT-F	TGCTCCCAGCCGAAGTTTGAAT	For real-time PCR of *HSP90* intron

HSP90-I-RT-R	TCTGAAGCTATTCAAGCTGCAACAAAG	
SBA1-I-RT-F	GGTGATTTCAAGCTTGATCGCATTTT	For real-time PCR of *SBA1* intron
SBA1-I-RT-R	AAAATTGTTGGCAGCGTAGGCG	
1391F	GTACACACCGCCCGTC	For real-time PCR of 18S rRNA
EukbR	TGATCCTTCTGCAGGTTCACCTAC	
RM2F	AGGGGCGAAAGACYAATCGAA	For real-time PCR of 28S rRNA
RM3R	CRCCAGTTCTGCTTACCAAAA	
SR6R	AAGWAAAAGTCGTAACAAGG	For real-time PCR of pre-rRNA
5.8S	CGCTGCGTTCTTCATCG	
TRI5-RT-F	GCCATTTTGGACCTTTCTGCTCATT	For real-time PCR of *TRI5*
TRI5-RT-R	GCCATAGAGAAGCCCCAACACAAT	
TRI6-RT-F	GGCAACCATTCAAGCGCTTTTTCT	For real-time PCR of *TRI6*
TRI6-RT-R	CACCCTGCTAAAGACCCTCAGACATT	
RPL16-spliced-RT-F	AGCTTCGAGCAAGTTGTCGTCATC	For real-time PCR of spliced and unspliced RNAs of *RPL16*
RPL16-spliced-RT-R	GAAGAACTCTCCGGAGATGTTAAGGG	
RPL16-unspliced-RT-F	CTCGTCGCTCGCCTTTCAGAT	
RPL28-spliced-RT-F	TCGAAGACCCGCAAGCACC	For real-time PCR of spliced and unspliced RNAs of *RPL28*
RPL28-spliced-RT-R	TGTTGGTACGGTGGTGGTGCT	
RPL28-unspliced-RT-F	=RPL28-I-RT-F	
RPL34-spliced-RT-F	CGGCTCCAAGCTCTCTGGTATCC	For real-time PCR of spliced and unspliced RNAs of *RPL34*
RPL34-spliced-RT-R	ACCGCATCGAGAACCACCGTAA	
RPL34-unspliced-RT-F	=RPL34-I-RT-F	
RPL36-spliced-RT-F	TGCTGGCCTCGCCCCCTA	For real-time PCR of spliced and unspliced RNAs of *RPL36*
RPL36-spliced-RT-R	CTTACGGGCACGCTTGTCCTT	
RPL36-unspliced-RT-F	=RPL36-I-RT-F	
miRTQ	CGAATTCTAGAGCTCGAGGCAGGCGAC	For reverse transcription of polyadenylated RNA[1]

	ATGGCTGGCTAGTTAAGCTTGGTACCGA GCTCGGATCCACTAGTCCTTTTT TTTTTTTTTTTTTTTTTTTTTVN	
RTQ-UNIr	CGAATTCTAGAGCTCGAGGCAGG	For real-time PCR of SnoRNAs (universal reverse primer)[1]
U18-F	CGTTTGACGAAGCAACAAGCACTT	For real-time PCR of U18
U24-F	CACGCAGGCCATGAGACCA	For real-time PCR of U24
SnR38-F	TCTCAAGAGTTCATTTGTGGATACCG	For real-time PCR of SnR38
SnR39-F	CTTCGGTCGATGCTGTTGATAGTTA	For real-time PCR of SnR39
SnR44-F	TCGGAATCCTTCTCATGATGTCTAA	For real-time PCR of SnR44
U18-I-RT-F	CGTTTGACGAAGCAACAAGCACT	For real-time PCR of U18-containing lariat
U18-I-RT-R	CGAGAGGGAAATGCGAAGGAAA	
U24-I-RT-F	CATTGCCCTGATCTTAACTTGCCAC	For real-time PCR of U24-containing lariat
U24-I-RT-R	AGCGACAAGGAAAATAAAGTTGGTTG	
SnR38-I-RT-F	GTCTAACTTCTGACTACGCAACCCCTC	For real-time PCR of SnR38-containing lariat
SnR38-I-RT-R	GGAAACAGAAAAAAACGGTGCCA	
SnR39-I-RT-F	GATTCTAATCATTTCTGCATCCACCTG	For real-time PCR of SnR39-containing lariat
SnR39-I-RT-R	TCGACCGAAGTCTAGTTGATGAAATGT	
SnR44-I-RT-F	GATAGATCCAAACTCGGAGACTTACACG	For real-time PCR of SnR44-containing lariat
SnR44-I-RT-R	ATTCGACGGCATATGGTGGTTG	

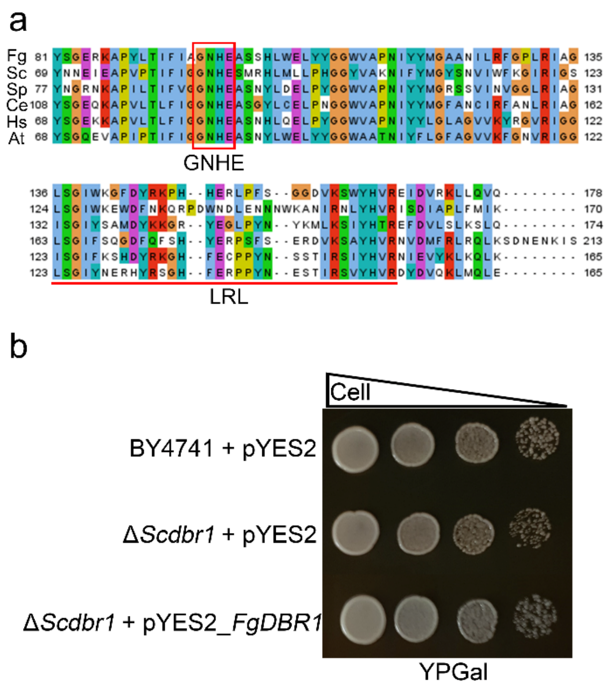

Supplementary Fig. 1 Sequence alignment of Dbr1 proteins and the phenotype of the *S. cerevisiae DBR1*-deletion mutant. **a** Multiple-sequence alignment of FgDbr1 orthologs. The alignment shows highly conserved GNHE motif and the lariat RNA recognition loop (LRL). Fg, *Fusarium graminearum*; Sc, *Saccharomyces cerevisiae*; Sp, *Schizosaccharomyces pombe*, Ce, *Caenorhabditis elegans*; Hs, *Homo sapiens*; At, *Arabidopsis thaliana*. **b** Growth phenotypes of *S. cerevisiae* strains. Cells of wild-type BY4741 containing the empty pYES2 vector and the mutant containing the empty pYES2 vector or pYES2-FgDBR1 were spot-inoculated on YPGal agar medium and incubated for 4 days at 30°C.

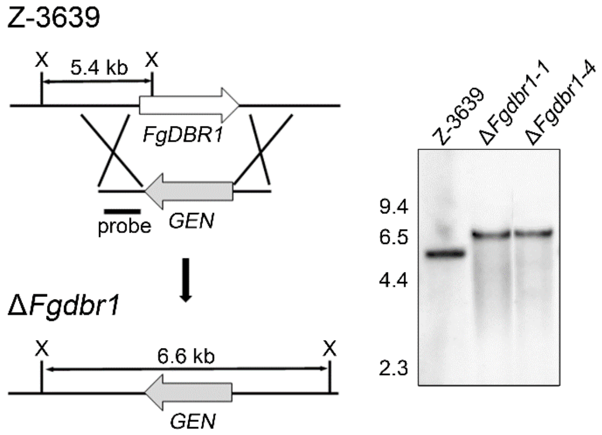

Supplementary Fig. 2 Targeted deletion of *FgDBR1*. Schematic illustration of the strategy used to delete *FgDBR1* (left panel) from the genome of the *F. graminearum* wild-type strain Z-3639. Southern blot analysis was used to confirm the deletion mutants (right panel). *GEN*, geneticin resistance gene cassette. The sizes of the DNA standards (kb) are indicated to the left of the blot. X, XhoI.

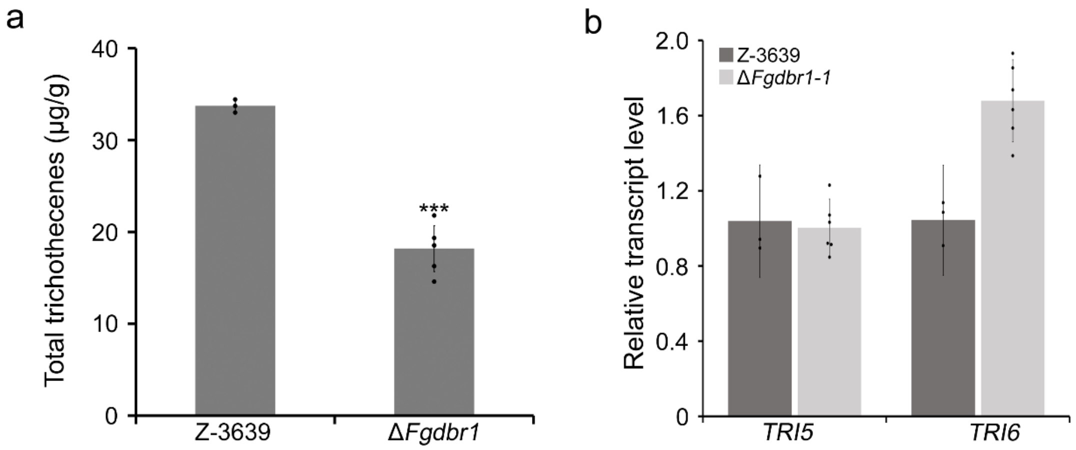

Supplementary Fig. 3 Trichothecene production of *F. graminearum* strains. **a** Total trichothecene (deoxynivalenol and 15-acetyldeoxynivalenol) production. Each strain was grown in minimal medium containing 5 mM agmatine (MMA) for 7 days. Trichothecene was analyzed using high-performance liquid chromatography and quantified relative to the biomass of each strain. **b** Transcript levels of *TRI5* and *TRI6* in the wild-type and Δ*Fgdbr1* strains. Transcript levels were quantified through quantitative real-time PCR (qRT-PCR) at 4 days after inoculation in MMA. Error bars represent standard deviations from three biological replicates. ***, $p < 0.001$.

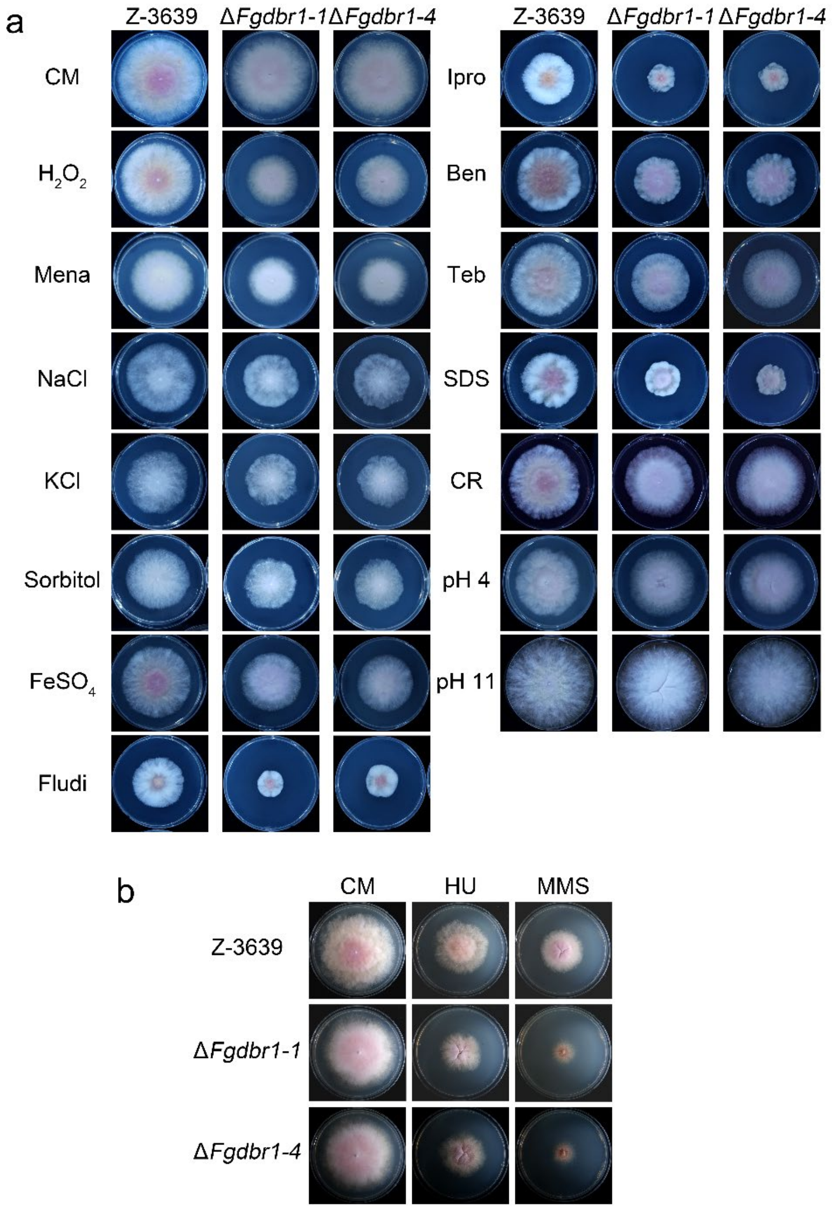

Supplementary Fig. 4 Mycelial growth of *F. graminearum* strains under various stress conditions. Images were taken at 5 days after inoculation. **a** Sensitivity of Δ*Fgdbr1* mutants on CM with different stress conditions. CM, complete medium; H_2O_2, 5 mM H_2O_2; Mena, 40 μM menadione; NaCl, 1 M NaCl; KCl, 1 M KCl; Sorbitol, 1.5 M sorbitol; $FeSO_4$, 4 mM $FeSO_4$; Fludi, 0.02 mg/L fludioxonil; Ipro, 7 mg/L iprodione; Ben, 0.65 mg/L benomyl; Teb, 0.025 mg/L tebuconazole; SDS, 100 mg/L sodium dodecyl sulfate; CR, 60 mg/L Congo red;

pH4 and pH11, CM of pH = 4 and pH = 11, respectively. **b** Sensitivity to DNA-damaging agents, 10 mM hydroxyurea (HU) and 0.1 μL/mL methyl methanesulfonate (MMS).

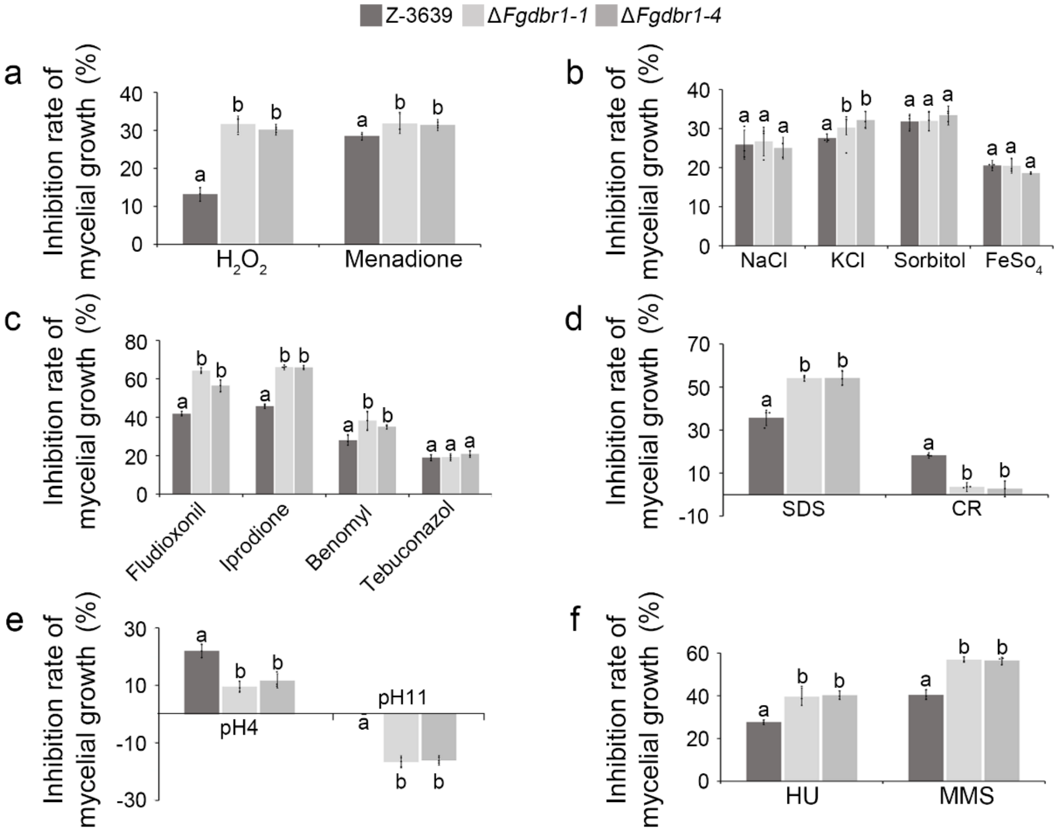

Supplementary Fig. 5 Inhibition rate of mycelial growth under various stress conditions. Percentage mycelial growth inhibition was examined at 5 days after inoculation on CM under the stress conditions described above (Supplementary Fig. 4). The tested conditions were oxidative stress **a**, osmotic stress **b**, fungicides **c**, cell-wall stress **d**, acidic or basic stress **e** and DNA-damage stress **f**. Error bars represent standard deviations from three biological replicates. Matching letters indicate no significant difference between strains, and different letters indicate statistically significant differences ($p < 0.01$).

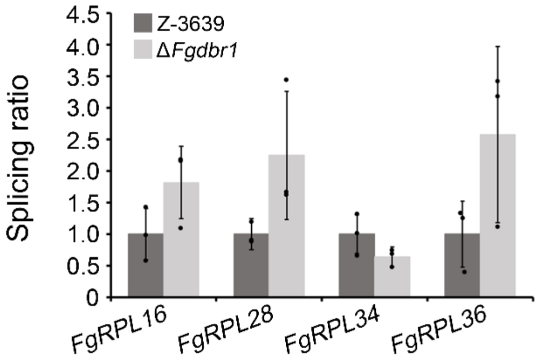

Supplementary Fig. 6 Splicing ratios of ribosomal protein genes. The splicing ratio was determined by calculating the level of spliced RNA normalized to the level of unspliced RNA for each intron using qRT-PCR analysis. The splicing ratio of the wild type was set to 1. Error bars represent standard deviations from three biological replicates.

Reference

1 Ro, S., Park, C., Jin, J., Sanders, K.M. & Yan, W. *Biochem. Biophys. Res. Commun.* **351**, 756-763 (2006).
