# Supplementary material for: Intron turnover is essential to the development and pathogenicity of the plant pathogenic fungus Fusarium graminearum
Source: Commun Biol. 2022 Oct 26;5:1129. doi: 10.1038/s42003-022-04111-3 (PMC9606315; doi:10.1038/s42003-022-04111-3)
Supplement: Supplementary file 2 — Description of Additional Supplementary Data [file 42003_2022_4111_MOESM2_ESM.docx]

**Description of Additional Supplementary Files**

**File name:** Supplementary Data 1

**Description:** List of up/down-regulated genes in FgDBR1 deletion mutant

**File name:** Supplementary Data 2

**Description:** List of genes included in GO:0005840 (ribosome)

**File name:** Supplementary Data 3

**Description:** List of F. graminearum strains used in this study

**File name:** Supplementary Data 4

**Description:** The source data behind the graphs in the paper
